# Rapid and Highly Selective Fe(IV) Generation by Fe(II)-Peroxyacid Advanced Oxidation Processes: Mechanistic Investigation via Kinetics and Density Functional Theory

**DOI:** 10.1021/acs.est.4c05234

**Published:** 2024-09-14

**Authors:** Junyue Wang, Juhee Kim, Jiaqi Li, Caroline Krall, Virender K. Sharma, Daniel C. Ashley, Ching-Hua Huang

**Affiliations:** † School of Civil and Environmental Engineering, Georgia Institute of Technology, Atlanta, Georgia 30332, United States; ‡ School of Public Health, Texas A&M University, College Station, Texas 77843, United States; § Department of Chemistry and Biochemistry, Spelman College, Atlanta, Georgia 30314, United States

**Keywords:** advanced oxidation processes (AOPs), density functional theory (DFT) calculations, high-valent iron, peroxyacids, Fenton-like

## Abstract

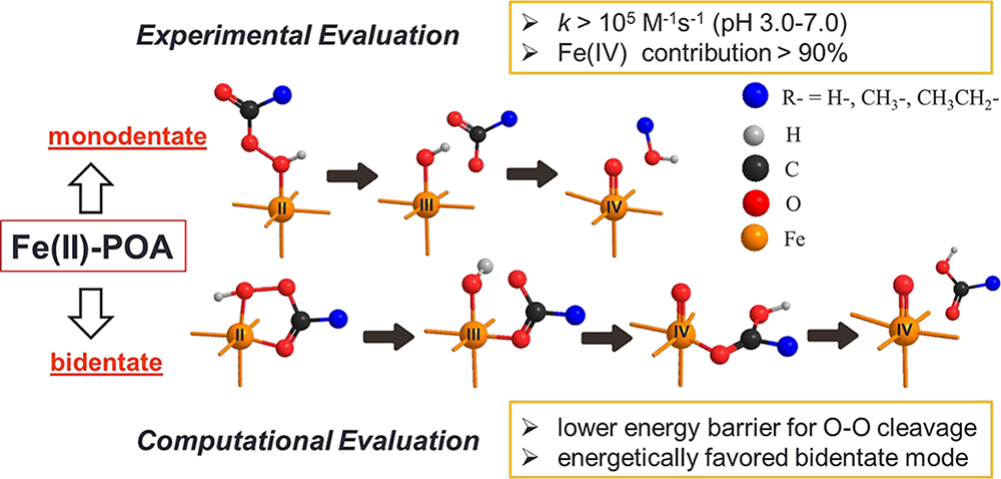

High-valent iron (Fe(IV/V/VI)) has been widely applied in water decontamination. However, common Fe(II)-activating oxidants including hydrogen peroxide (H_2_O_2_) and persulfate react slowly with Fe(II) and exhibit low selectivity for Fe(IV) production due to the cogeneration of radicals. Herein, we report peroxyacids (POAs; R–C(O)OOH) that can react with Fe(II) more than 3 orders of magnitude faster than H_2_O_2_, with high selectivity for Fe(IV) generation. Rapid degradation of bisphenol A (BPA, an endocrine disruptor) was achieved by the combination of Fe(II) with performic acid (PFA), peracetic acid (PAA), or perpropionic acid (PPA) within one second. Experiments with phenyl methyl sulfoxide (PMSO) and *tert*-butyl alcohol (TBA) revealed Fe(IV) as the major reactive species in all three Fe(II)-POA systems, with a minor contribution of radicals (i.e., ^•^OH and R–C(O)O^•^). To understand the exceptionally high reactivity of POAs, a detailed computational comparison among the Fenton-like reactions with step-by-step thermodynamic evaluation was conducted. The high reactivity is attributed to the lower energy barriers for O–O bond cleavage, which is determined as the rate-limiting step for the Fenton-like reactions, and the thermodynamically favorable bidentate binding pathway of POA with iron. Overall, this study advances knowledge on POAs as novel Fenton-like reagents and sheds light on computational chemistry for these systems.

## Abstract 2

This paper systematically studied the Fenton-like reactions of three different peroxyacids (performic acid, peracetic acid, and perpropionic acid) and elucidated their high reactivity from experimental and computational perspectives.

## Introduction

Fe(II) activation of dioxygen and peroxides plays an essential role in natural environments and living cells.^1−5^ The Fenton-like reactions between Fe(II) and peroxides generate highly oxidative species, including high-valent iron and/or hydroxyl radicals, which can initiate advanced oxidation processes (AOPs).^6^ These reactions have been extensively harnessed for organic synthesis,^7−9^ bioinspired synthesis of enzyme-like iron complexes,^10−12^ chemodynamic therapy,^13,14^ and water purification.^15−18^ Fenton-like reactions usually successively undergo (1) peroxide complexation with iron, (2) O–O bond breakage, and (3) hydrogen atom transfer (HAT),^19^ where the feasibility of HAT determines the major reactive species formed in the system (Fe(IV) versus radicals). Hydrogen peroxide (H_2_O_2_)^7,17^ and persulfate (S_2_O_8_^2–^)^20,21^ are common oxidants in Fenton-like systems, but they react rather slowly with Fe(II) (second-order rate constants *k* < 80 M^–1^ s^–1^, pH 3.0–7.0) and suffer a decrease in decontamination efficiency at neutral pH values.^2,21^ Furthermore, these systems generate radicals and Fe(IV) at comparable levels, where the radicals can be easily scavenged by environmental background constituents, leading to significantly lowered AOP performance.^2,21^

**Figure 1:**
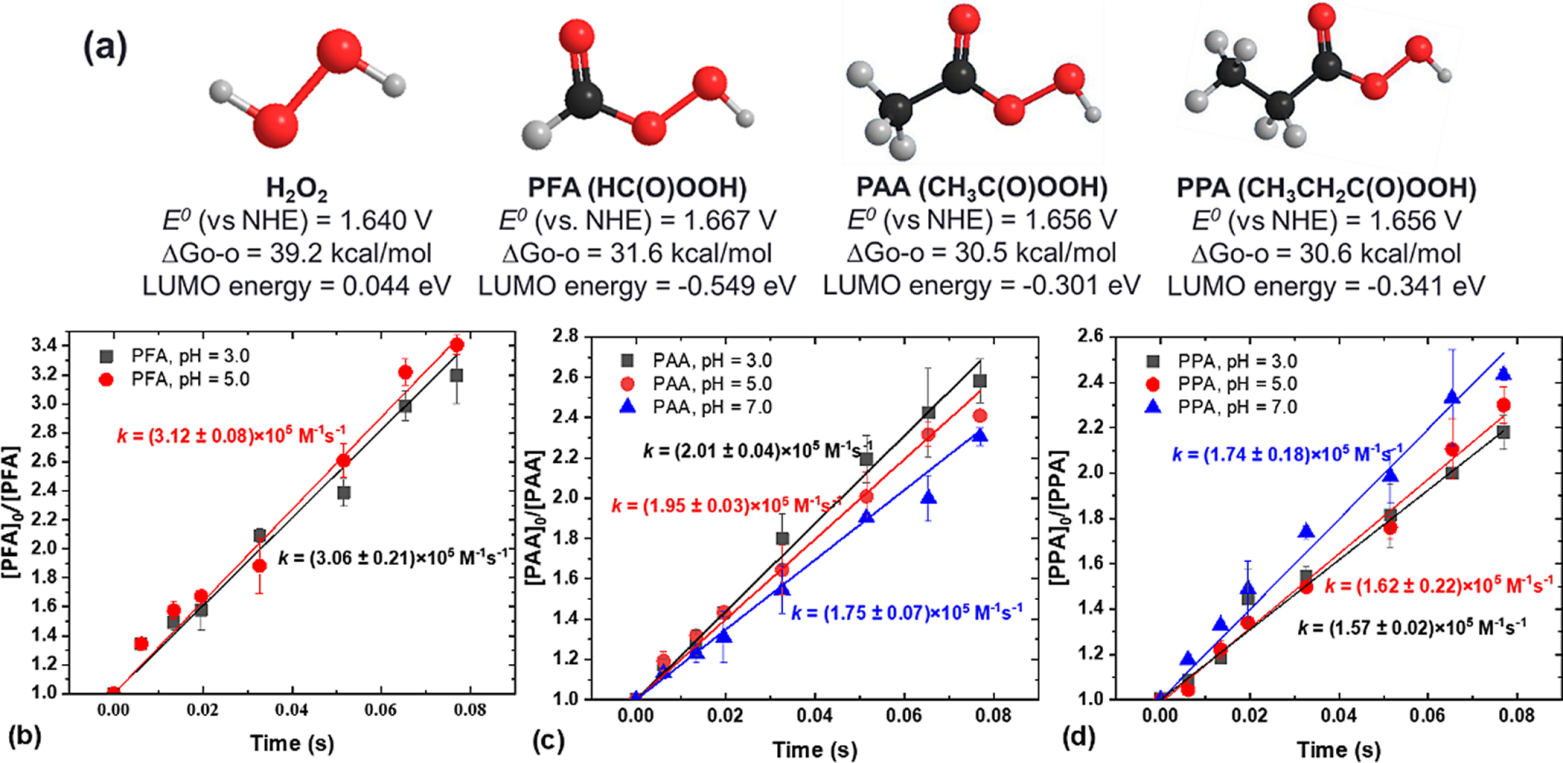
Structures of peroxides and their DFT-calculated chemical properties (a) and degradation of POAs (b–d) in Fe(II)-POA processes. Experimental conditions: [POA]_0_ = [Fe(II)]_0_ = 100 μM, [coexistent H_2_O_2_]_0_ = 57–63 μM, and no buffer.

Recently, peroxyacids (POAs, Figure 1) have emerged as novel oxidants for food disinfection^15,22^ and wastewater treatment^23,24^ due to their pathogen inactivation efficacy and lower byproduct formation potentials.^24−28^ Peracetic acid (PAA), a representative POA with commercial products, has been extensively studied in Fenton-like AOPs, with Fe(II)/Fe(III),^29,30^ Co(II)/Co(III),^31−34^ Ru(III),^35^ and Mn(II)^36^ as metal activators, with or without additional chelating agents. Among the above-cited studies, Fe(II)-PAA achieved the most rapid degradation of organic contaminants (<1 s) due to the fast activation reactions (Table 1).^30^

**Table 1:** Reactions in Fe(II)-POA Systems and Their Second-Order Rate Constants

no.	reactions	*k* (M^–1^ s^–1^)	ref
Fe(II)-activation of POAs			
R1	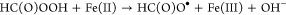	overall: (3.06–3.12) × 10^5^ (pH 3.0, 5.0)	this study
R2	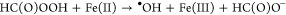		this study
R3	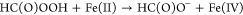		this study
R4	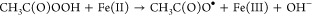	overall: (1.75–2.01) × 10^5^ (pH 3.0, 5.0, 7.0)	this study
R5	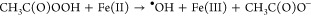		this study
R6	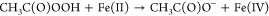		this study
R7	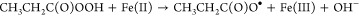	overall: (1.57–1.74) × 10^5^ (pH 3.0, 5.0, 7.0)	this study
R8	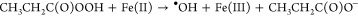		this study
R9	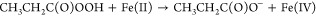		this study
Formation and fate of organic radicals			
R10	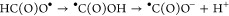	fast	(42)
R11	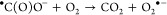	2.0 × 10^9^	(42)
R12	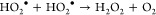	8.3 × 10^5^	(44)
R13	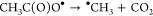	1.0 × 10^5^ s^–1^	(44)
R14	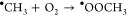	(2.8–4.1) × 10^9^	(44)
R15	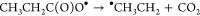		(45,46)
R16	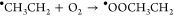		(46)
R17	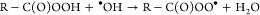	∼1 × 10^9^	(44)
	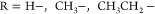		
R18	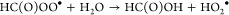	2.46 × 10^3^ s^–1^	(41,42)
R19	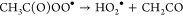	1.82 s^–1^	(44)
R20	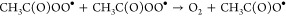	8.3 × 10^9^	(44)
R21	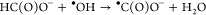	2.1 × 10^9^	(31)
R22	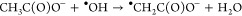	8.5 × 10^7^	(30)
R23	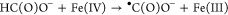	7.5 × 10^4^ (pH 3.0)	(47)

Performic acid (PFA, HC(O)OOH) and perpropionic acid (PPA, CH_3_CH_2_C(O)OOH) are the two simplest homologues of PAA, with only one less methyl group and one more methyl group than PAA, respectively. Therefore, the combination of Fe(II) with PFA and PPA may initiate similar Fenton-like reactions (Table 1) and achieve highly efficient abatement of micropollutants as PAA. Nonetheless, the feasibility and mechanisms of PFA and PPA in Fenton-like AOPs have never been studied due to a lack of commercial availability. Moreover, the high reactivity of POAs with aqueous Fe(II) has never been clearly understood and illustrated from thermodynamic perspectives.

Therefore, the objectives of this study were to (1) revisit the reaction kinetics between the three POAs and Fe(II) (without additional chelating agents present) using a KinTek quenched flow system (QFS-1) that allows a reaction time as low as 0.006 s; (2) compare the reactivity, decontamination efficiency, and reactive species in the three Fe(II)-POA AOPs; and (3) conduct density functional theory (DFT) calculations to provide a step-by-step mechanistic evaluation to better understand the high reactivity of POAs with Fe(II).

## Methods and Materials

### Chemicals and Reagents

The sources of all used chemicals and reagents and the methods for POA synthesis are detailed in Text S1.

### Batch Experiments

One organic contaminant was mixed with the selected POA in a 50 mL glass beaker. Then, the POA concentration was measured, and the pH was immediately adjusted to the desired value by the addition of small drops of sulfuric acid (H_2_SO_4_) and sodium hydroxide (NaOH). Then, a freshly prepared stock solution of Fe(II) (dissolved FeSO_4_) was dosed to initiate the reaction. The reaction solution was magnetically stirred and opened to the ambient air. A one milliliter sample (1 mL) was taken after 10 s of the reaction time and quenched by excess Na_2_S_2_O_3_ for the measurement of contaminant. Blank control experiments (without any metals) showed that contaminant loss due to adsorption onto the beakers, volatilization, and photolysis by ambient light, and oxidation by POA alone was minimal (data not shown).

### Quenched Flow System Experiments

The rapid reaction experiments were conducted using a KinTek quenched flow system (named QFS-1) (KinTek corp., Snow Shoe, PA, USA) or a custom-built QFS setup (named QFS-2) similar to that by Kim et al.^30^ The retention time accuracy of the KinTek system was validated by the reaction between hypoiodous acid (HOI) and bisphenol A (BPA) (Figure S1).

Briefly, solutions of Fe(II) (200 μM, with or without an organic contaminant) and POA (200 μM, pH adjusted by H_2_SO_4_ and NaOH) were prepared in two syringes connected to capillary tubing, respectively. Each of the quenched flow systems was precisely pumped, and the two solutions were rapidly mixed. After designated time intervals (e.g., achieved by adjusting the length of tubing in the QFS), the reaction was quenched by excess KI, ferrozine, or Na_2_S_2_O_3_ from a third syringe, which allowed measurement of POAs, Fe(II), or micropollutants, respectively. The QFS-1 had a flow rate of 156.0 mL/min and could achieve a mixing time from 0.006 to 0.077 s, while QFS-2 had a flow rate of 10.5 mL/min that resulted in a mixing time from 0.152 to 1.080 s (depending on the capillary tubing length).

### Analytical Methods

The concentrations of POAs and Fe(II) were measured using potassium iodide-*N,N*-diethyl-*p*-phenylenediamine (KI-DPD) and ferrozine methods, respectively, on a UV–vis spectrophotometer as detailed in our previous study.^37^ Note that preliminary experiments confirmed ferrozine complexation could suppress the reaction between Fe(II) and PAA. The concentrations of four target compounds (i.e., bisphenol A (BPA), benzoic acid (BA), methyl phenyl sulfoxide (PMSO), and methyl phenyl sulfone (PMSO_2_)) were analyzed using an Agilent 1100 high-performance liquid chromatograph equipped with an Agilent Zorbax SB–C18 column (2.1 × 150 mm, 5 μm) and a diode-array detector (HPLC-DAD) (Table S1).

### Density Functional Theory Calculations

All electronic structure calculations were performed using density functional theory (DFT) as implemented in the Gaussian 16 electronic structure package.^38^ Details of computational chemistry methods are provided in Text S2.

## Results and Discussion

### Reaction Kinetics of Fe(II)-POAs

First, we quantified the rate constants (*k*) between POAs and Fe(II) using a KinTek quenched flow system (QFS-1) by mixing POA and Fe(II) at equimolar amounts and measuring the POA loss on a UV–vis spectrophotometer.^37^ It is noteworthy that POA solutions contain H_2_O_2_ and the corresponding carboxylic acid (Text S1). However, H_2_O_2_ reacts with Fe(II) slowly (*k* = 63 M^–1^ s^–1^ at pH 3.0).^39,40^ In our previous study, we also demonstrated that the side reaction of Fe(II)-H_2_O_2_ was negligible within the time scale of this study (up to 1.08 s for QFS and 10 s for batch reactors).^30^ Furthermore, we found that reactive species generation from Fe(II)-H_2_O_2_ in the presence of carboxylic acids (formic acid, acetic acid, or propionic acid) was negligible within 30 s (Figure S2). In addition, the self-decay of all three POAs was negligible within the reaction time in this study (Figures S3 and S4).

To determine the second-order rate constants between Fe(II) and POAs, PMSO was dosed in the reaction as a scavenger to minimize the contributions of Fe(IV) and radicals to POA degradation, as PMSO is known to react efficiently with both Fe(IV) and ^•^OH.^21,30,31^ However, 500 μM PMSO had little impact on POA degradation (Figure S5), indicating a minor contribution of Fe(IV) and ^•^OH to POA degradation and confirming Fe(II) as the major reactant for POA degradation. Furthermore, Fe(II) loss was measured in the Fe(II)-PAA system at three pH values to confirm the stoichiometry for Fe(II)-POA reactions. As shown in Figure S6, Fe(II) was consumed with a near 1:1 stoichiometry with PAA. It should be noted that Fe(II) consumption was slightly slowed at the end of the reaction compared with PAA, suggesting that reactive species (e.g., Fe(IV) and radicals) may play a minor role in degrading PAA. Considering that Fe(II) reacts with POA at a 1:1 molar ratio (Table 1, R1–R9), Fe(II) and POA concentrations can be fitted to a second-order kinetic model (eq 1), which can be converted to eq 2 by integration over time.
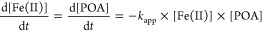
1
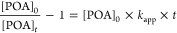
2where *k*_app_ is the apparent second-order rate constant (in M^–1^ s^–1^), *t* is the reaction time (in s), and [Fe(II)] and [POA] are the concentrations (in M) of Fe(II) and POA. The results showed that PAA and PPA reacted with Fe(II) at the apparent second-order rate constant (*k*_app_) = (1.57–2.01) × 10^5^ M^–1^ s^–1^ (pH 3.0–7.0), and pH did not affect the reaction rate constant significantly (Figure 1b–d). However, note that the rate constants were reported at their initial pH values, and the solution was not buffered to avoid any potential complexation of the Fe species. As significant pH drops were observed during the reactions (especially for pH 7.0, Table S2), the measured rate constants could represent the values only at the initial pH conditions rather than at stable constant pH values. The reaction between PFA and Fe(II) was even faster at *k*_app_ = (3.06–3.12) × 10^5^ M^–1^ s^–1^ (pH 3.0 and 5.0), about 4 orders of magnitude faster than that of H_2_O_2_ (*k*_app_ = 63 M^–1^ s^–1^ at pH 3.0).^39^

### BPA Oxidation by Fe(II)-POA AOPs

**Figure 2:**
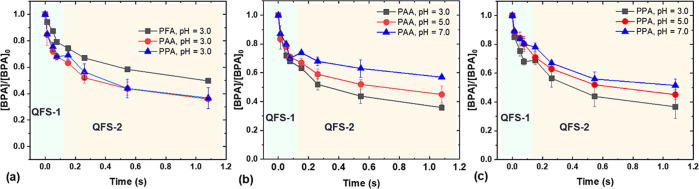
BPA degradation (a–c) by Fe(II)-POAs at different pH values. Experimental conditions: [BPA]_0_ = 10 μM, [POAs]_0_ = [Fe(II)]_0_ = 100 μM, [coexistent H_2_O_2_]_0_ = 57–63 μM, and no buffer.

BPA, a common micropollutant that reacts with both Fe(IV) and ^•^OH, was employed to test the efficiency of the Fe(II)-POA AOPs. The rapid degradation of BPA by Fe(II)-POA processes was studied using both QFS systems (QFS-1 and QFS-2). At pH 3.0, more than 60% of BPA was degraded within only 1.080 s in both the Fe(II)-PAA and Fe(II)-PPA systems (Figure 2a). Interestingly, although PFA reacted faster with Fe(II), Fe(II)-PFA was not as efficient as Fe(II)-PAA and Fe(II)-PPA for degrading BPA due to the significant scavenging effect of coexistent formic acid (see later discussion).

### Reactive Species in Fe(II)-POA AOPs

**Figure 3:**
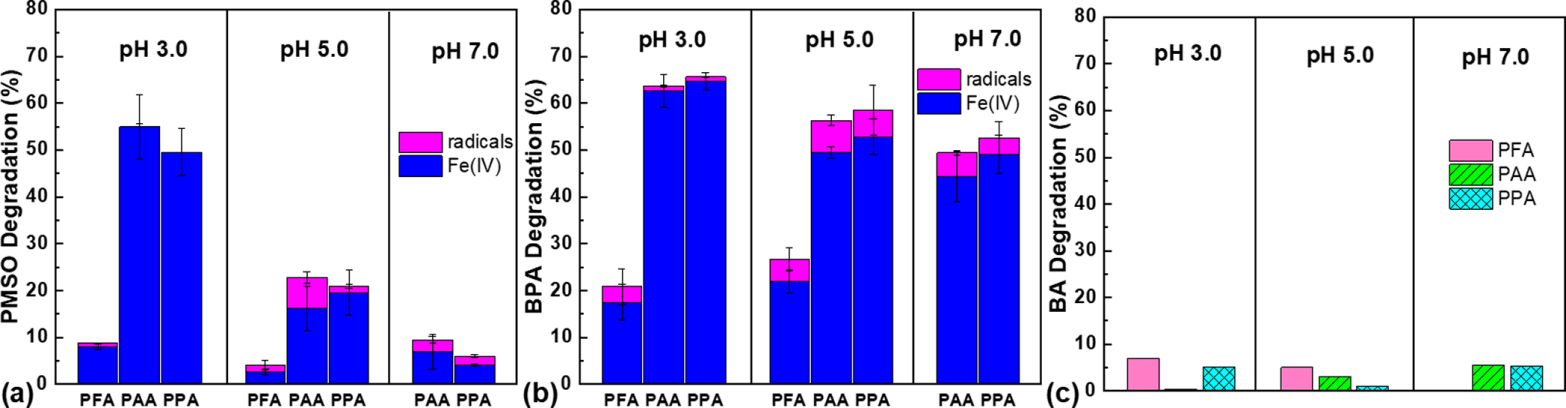
Contributions of Fe(IV) and radicals to PMSO (a), BPA (b), and BA (c) degradation by Fe(II)-POAs at 10 s. Experimental conditions: [POAs]_0_ = [Fe(II)]_0_ = 100 μM, [coexistent H_2_O_2_]_0_ = 57–63 μM, and no buffer. Where applicable, [BPA]_0_ = 10 μM, [PMSO]_0_ = 50 μM, [TBA]_0_ = 50 mM, and [BA]_0_ = 25 μM.

The initial reaction between Fe(II) and POAs could produce ^•^OH, Fe(IV), or acyloxyl radicals (R–C(O)O^•^) (Table 1, R1–R9). However, the acyloxyl radicals usually undergo rapid decay (Table 1, R10–16) and hence could not efficiently contribute to BPA degradation. In addition, the chain reaction with POA could produce acylperoxyl radicals as secondary reactive species (Table 1, R17). Formylperoxyl radical undergoes fast hydrolysis (Table 1, R18),^41,42^ while acetylperoxyl radical is relatively stable and may contribute to the degradation of organic contaminants. To delineate the relative contributions of Fe(IV), hydroxyl radical or acylperoxyl radical (particularly in Fe(II)-PAA), PMSO, *tert*-butyl alcohol (TBA), and benzoic acid (BA) were applied as probes/scavengers. It is well documented that PMSO_2_ is the only oxidation product of PMSO by Fe(IV), while radical (e.g., ^•^OH and organic radicals) oxidation of PMSO does not lead to PMSO_2_.^21,37^ Thus, PMSO_2_ production indicates the contribution of Fe(IV) to PMSO oxidation, while other PMSO loss can be attributed to radicals (Table S3). As shown in Figure 3a, Fe(IV) dominated PMSO removal at all pH values, while the radicals only had minor contributions at pH 5.0 and 7.0. Notably, acetylperoxyl radical has low reactivity with PMSO, and hence, PMSO conversion could not prove the absence of acetylperoxyl radical.^37^

TBA is recognized as a selective quencher for ^•^OH, with no obvious influence on Fe(IV).^30,43^ Moreover, TBA is also expected to inhibit the generation of organic peroxyl radicals (R–C(O)OO^•^) by quenching their precursor (i.e., ^•^OH, Table 1 R17).^44^ Although TBA may not react with acyloxyl radicals, those radicals undergo fast self-decay and their contribution to BPA degradation should be minimal.^42,44−46^ Thus, the BPA removal that could not be quenched by TBA was attributed to Fe(IV) oxidation, while the quenched part was attributed to radicals (Table S4). As shown in Figure 3b, the contribution of radicals to BPA removal by Fe(II)-POA processes was minor to minimal at all pH values, further confirming Fe(IV) as the major reactive species (Table 1 R3, R6, and R9).

BA is reactive with organic radicals (e.g., CH_3_C(O)O^•^ and CH_3_C(O)OO^•^) and ^•^OH, while it exhibits sluggish reactivity toward Fe(IV) (*k* < 80 ± 20 M^–1^ s^–1^ at pH 0).^37,47^ We found that BA removal by Fe(II)-POAs at all tested pH values was minimal (<5%) (Figure 3c). Thus, unlike other Fenton-like reactions, Fe(II)-POA processes selectively generate Fe(IV) rather than radicals in a wide pH range (3.0–7.0).

### pH Effects

pH plays a critical role in Fenton-like AOPs by driving the speciation of both metal activators and oxidants. Fe^2+^, Fe(OH)^+^, and Fe(OH)_2_(aq) are major species of Fe(II) at pH < 7.0, and their reaction kinetics as reducing agents follow the order: Fe^2+^ < Fe(OH)^+^ < Fe(OH)_2_.^2^ Thus, with increasing pH, the apparent second-order rate constants between Fe(II) and the oxidants should increase due to the speciation from Fe^2+^ to Fe(OH)^+^ and Fe(OH)_2_(aq), which in turn accelerates the generation of reactive species. However, Fe(II) also competes with micropollutants for reactive species, and hence, the scavenging of reactive species is also enhanced by the increasing pH.^48^

As shown in Figure 2b,c, BPA degradation by Fe(II)-PAA and Fe(II)-PPA was mildly inhibited by increasing the initial pH (pH dropped during the reaction; see Table S2). The reported p*K*_a_ values for PFA and PAA were 7.3 and 8.2, respectively.^24,49^ The p*K*_a_ value for PPA is not available but is expected to be slightly higher than that of PAA. Hence, all three POAs were mainly in their protonated forms in these experiments. Moreover, as the reactions between Fe(II) and PAA/PPA were negligibly decreased by the increase in pH (Figure 1b–d), the generation of reactive species should remain at high levels as the pH increases. Thus, the slight inhibition of BPA removal could be caused by the (i) faster scavenging and/or self-decay of reactive species^2,18^ and (ii) the potential decrease of the oxidation capacity of reactive species (e.g., Fe(IV)) at higher pH values.^16,50^

^•^OH has a p*K*a at 11.8–11.9 and hence will always stay in its protonated form within the pH range of 3.0–7.0. Thus, the variation of its self-decay rate and oxidation capacity should be minimal in the pH range of this study. Furthermore, it contributed negligibly to the oxidation of micropollutants (Figure 3). Therefore, the slightly inhibited oxidation at higher pH values should be attributed to enhanced self-decay and/or loss of oxidation capacity of Fe(IV), which was the predominant reactive species in all three Fe(II)-POA systems (Figure 3).

In the absence of biomimetic chelating agents, Fe(IV) will be mainly in its hydroxyl-complexed forms that easily decompose with a lifetime <0.5 s at pH 3.0.^51^ So far, research on Fe(IV) decay and its scavenging reactions has been limited to only extreme pH conditions (e.g., pH 0, 10.0) due to the challenges to characterize the transient Fe(IV) species. To gain mechanistic insights into the oxidation efficiency loss due to side-reactions of Fe(IV), the original studies reporting Fe(IV) decay rates at various pH values are summarized in Table 2. In brief, Fe(IV) could be consumed by hydrolysis (Table 2 R1, R2), H_2_O_2_ (Table 2 R3), Fe(II) (Table 2 R4), and bimolecular decay (Table 2 R5), where R2 is usually a negligible pathway compared with the other reactions.^48^ As we confirmed that Fe(II) was mainly consumed by POAs rather than Fe(IV), we expect that R4 (Table 2) should provide a minimal contribution to Fe(IV) consumption under our experimental conditions. The coexistent H_2_O_2_ could be an important consumer for Fe(IV); however, this reaction seems to be not sensitive to pH, hence contributing constantly in the pH range 3.0–7.0 (Table 2). Therefore, the decreased oxidation efficiency at circumneutral pH values should be attributed to the accelerated Fe(IV) consumption through R1 and/or R5 (Table 2) and the potential decrease in its oxidation efficiency due to a pH increase. The bimolecular Fe(IV) decay (R5) has been studied across a wide pH range, and its rate constant increases by ∼5 orders of magnitude with an increasing pH from 0 to 10.0, accounting for the markedly accelerated Fe(IV) decay at higher pH values. On the other hand, the significance of Fe(IV) hydrolysis reaction (R1) has only been reported at pH 1.0 and is worthy of further investigation.

**Table 2:** Fe(IV) Self-Decay Reactions and their Second-Order Rate Constants

no.	reactions	rate constants (M^–1^ s^–1^ or s^–1^)				
		pH 0	pH 1	pH 3	pH 7	pH 10
R1	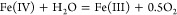		0.1			
R2	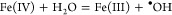	1.3 × 10^–2^	2.5 × 10^–2^	∼1		<0.5
R3	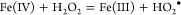	1 × 10^4^	1 × 10^4^		1 × 10^4^	3.9 × 10^5^
R4	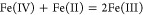	1.4 × 10^5^	4.33 × 10^4^			1 × 10^6^
R5	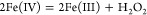	<50			1 × 10^3^	8 × 10^5^
ref		(47)	(48,51)	(51)	(68,69)	(70)

### Scavenging Effects of Coexistent Carboxylic Acids

POA synthesis utilizes the reactions between H_2_O_2_ and the carboxylic acids (R–C(O)OH + H_2_O_2_ ↔ R–C(O)OOH + H_2_O).^24,30,52^ Therefore, PFA, PAA, and PPA solutions contain formic acid (FA), acetic acid (AA), and propionic acid (PA), respectively. These carboxylic acids may act as scavengers for reactive species (e.g., Fe(IV) and ^•^OH) and lower the performance of the AOPs. For example, FA reacts with ^•^OH to produce ^•^CO_2_^–^ and subsequently HO_2_^•^ (Table 1 R21, 11),^42,53^ whereas the reaction between AA and ^•^OH produces ^•^CH_2_COO^–^ (Table 1 R22).^44^ These daughter radicals are not useful for contaminant degradation due to lower reactivity with organic compounds.^44,54−56^ However, FA is a more significant scavenger due to the susceptibility of the formaldehyde moiety to oxidation. For example, the second-order rate constants with ^•^OH for protonated FA and AA were reported to be 2.1 × 10^9^ and 8.5 × 10^7^ M^–1^ s^–1^, respectively.^25,57^ Besides ^•^OH, Fe(IV) is also a major reactive species in Fenton-like systems.^2,21,30^ The reactivity of carboxylic acids with Fe(IV) has been studied at pH 0–3.0 by Jacobsen et al., where the rate constants between FA with Fe(IV) is ∼2 orders of magnitude higher than that of AA (Table 1 R23).^47^ Nevertheless, the reaction rate constants of Fe(IV) with FA, AA, and PA have not been reported at neutral pH values. To delineate the scavenging effect of FA, AA, and PA in the Fe(II)-POA systems, we spiked additional FA, AA, and PA into the Fe(II)-PAA system to compare their effects on BPA removal, where Fe(IV) was confirmed to be the major reactive species. As shown in Figure S7, the inhibitory effect of FA was much more significant than AA and PA for the abatement of BPA by the Fe(II)-PAA system at all tested pH values (3.0–7.0), confirming that the coexisting FA in PFA solution hindered BPA degradation in the Fe(II)-PFA system. However, it is noteworthy that BPA degradation cannot be completely inhibited by 1.0 mM FA, indicating a high selectivity of Fe(IV) to react with phenolic compounds rather than carboxylic acids.^58−60^

In addition to the scavenging by FA, the relatively low efficiency of Fe(II)-PFA may also be attributed to the generation of nonreactive organic radical (Table 1 R1), which was not useful for BPA degradation and mitigated the electron utilization efficiency. It should be noted that this assumption could not be verified using the employed experimental setup and is beyond the scope of the current study.

### Density Functional Theory Calculations

**Figure 4:**
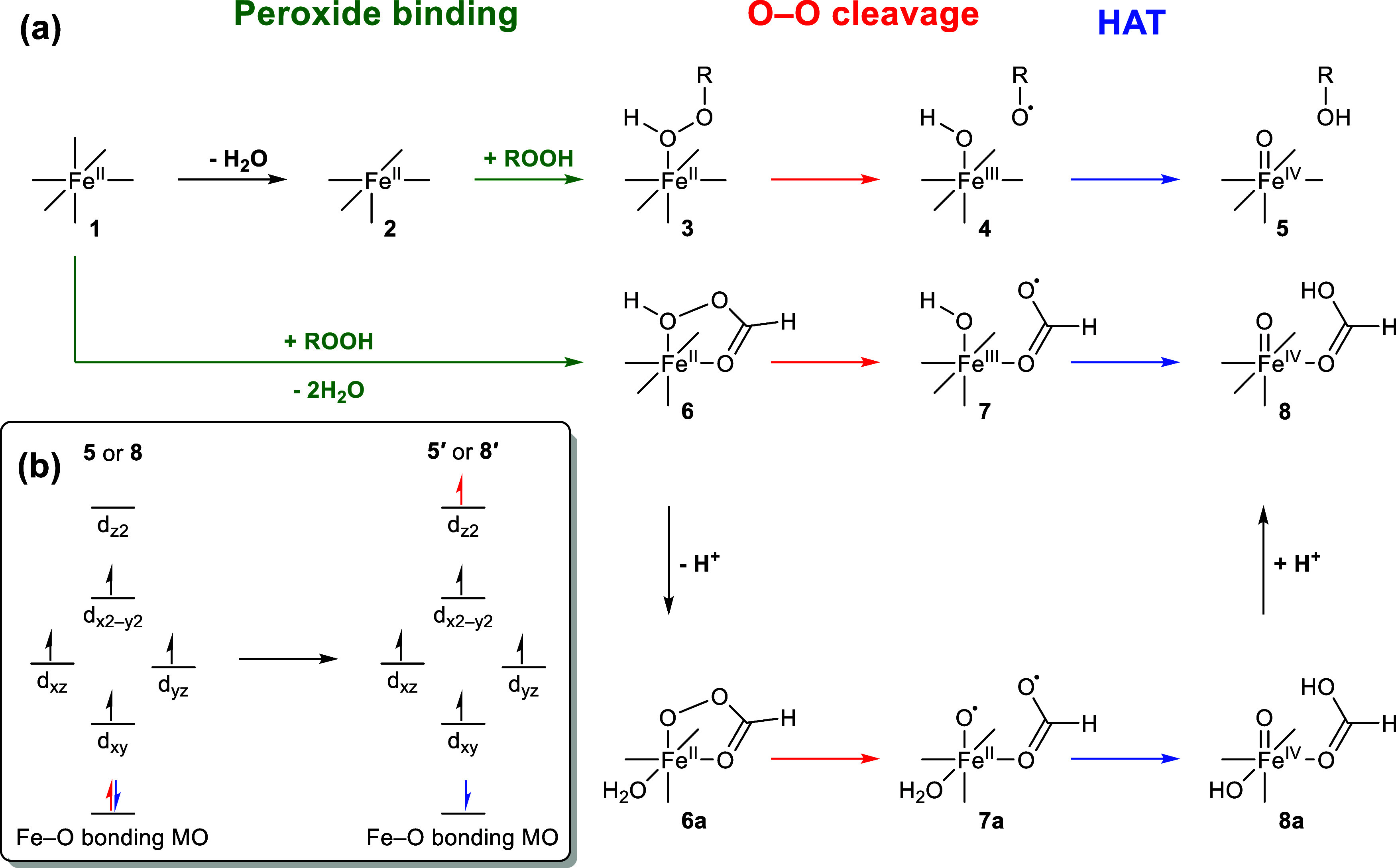
Calculated mechanisms for Fe(II)-peroxide (a) and simplified molecular orbital diagrams of the electronic structures calculated for the Fe(IV) species (b).

DFT calculations were used to model potential mechanisms for Fe(II) oxidation by H_2_O_2_ and POAs (Text S2), with a previous DFT study by Lu et al. on Fe(II)-H_2_O_2_ serving as a starting point.^19^ Lu’s study was able to successfully rationalize the experimental observation that the reactive oxidants during the Fenton reaction are ^•^OH and Fe(III) at low pH and Fe(IV) at higher pH (above 2.2, the p*K*_a_ of aqueous Fe(III)). The mechanism based on their calculations began with [Fe(H_2_O)_5_(H_2_O_2_)]^2+^, which could undergo O–O homolysis to form [Fe(H_2_O)_5_(OH)]^2+^ with an incipient ^•^OH hydrogen bonded to the bound water molecules. This intermediate could either reform the starting hydroperoxide complex or ^•^OH could abstract a hydrogen atom to form water and Fe(IV). We sought to see if a similar mechanism could be calculated with reasonable barrier heights when POAs were used instead and to also compare these barrier heights to those calculated using H_2_O_2_ (Figure 4a). These results are briefly described below and in more detail in the Supporting Information (Text S2–S4).

The exchange of an aqua ligand for a peroxide on [Fe(H_2_O)_6_] (**1**) was always found to be slightly energetically uphill (Table S5). Before the evaluation of the O–O bond cleavage, the protonation states of the resulting metal-bound peroxides (**3**) were evaluated first. It is well-known that calculating p*K*_a_s can be very difficult to perform accurately, and in this case, we did so using the same methodology as Lu and co-workers, where effectively experimentally known p*K*_a_s were used to generate a calibration curve (Text S2).^19^ These results (Table S6) indicated that **3** would be predominantly protonated at pH = 3.0 for all examined peroxides, and therefore, potential mechanisms involving monodentate deprotonated peroxide complexes were not explored further.

When considering the binding of POAs to a metal, it was important to also evaluate their ability to act as chelating ligands. Unlike H_2_O_2_, POAs can have unique coordination through the terminal peroxide oxygen and the carbonyl oxygen simultaneously to form a five-membered ring (Figure 4a). Similar chelated structures have been shown for nonheme iron complexes as well, through both the reaction of these Fe(II) complexes with a POA or the combination of a carboxylic acid and H_2_O_2_.^10,46,61^ In these cases, the resulting chelated intermediate formed is in the Fe(III) state, which can then lead to a formal Fe(V) oxidant. In the present study, we considered a different mechanism, namely, the direct reaction of POA bound to Fe(II) to produce an Fe(IV) oxidant. Although it is possible that Fe(III) can be formed in our system and then subsequently react with the POAs, we have shown previously that PAA reacts much more slowly with Fe(III) than Fe(II) under these conditions, and so the reaction with Fe(II) is more relevant.^30^ Another important difference from these previous works on nonheme Fe complexes is that, although they are of course quite relevant to the present study, these previous complexes had significantly different ligand environments, as the Fe centers were supported by multidentate polyamine ligands, as opposed to simply aqua ligands in the present case.

The calculated results showed that the bidentate binding mode was slightly favored over the monodentate binding mode (Table S5). Enthalpically, it is less favorable (due to the lower Lewis basicity of the carbonyl oxygen compared to a water molecule); however, the chelate effect confers an entropic advantage, making both binding modes reasonable. Calculations of p*K*_a_ indicated that of all the bidentate bound structures, only for the PFA-bound bidentate structure is the deprotonation predicted to be significant at pH 3.0 (p*K*_a_ = 3.2). However, the deprotonated energetics (thermodynamics and barrier heights) were not significantly different from the protonated energetics regardless (Tables S5 and S6), and so, discussion of this pathway is left to the SI.

Transition states (TSs) were located for O–O bond cleavage for the POAs, with each resulting in a carboxyl radical fragment antiferromagnetically coupled (opposite spins on each fragment) to a (formally) high-spin Fe(III)–OH, analogous to what was seen for H_2_O_2_,^19^ The resulting barrier heights (Δ*G*^‡^) were very reasonable for reactions that should occur readily at room temperature and ranged from 8.0 to 10.0 kcal·mol^–1^ for the POAs and 13.6 kcal·mol^–1^ for H_2_O_2_ (Table S5). The larger barrier height for H_2_O_2_ almost certainly originates from the fact that it has a stronger O–O bond: the bond dissociation enthalpy for H_2_O_2_ was calculated as 46.0 kcal·mol^–1^, while for the POAs, it was calculated as 40.5, 39.9, and 39.8 kcal·mol^–1^ for PFA, PAA, and PPA, respectively (Figure 1a shows a similar trend in the Δ*G* values for O–O homolysis). Interestingly, the cleavage barrier heights did not show a large dependence on protonation or binding mode, with all being comparable (Table S5).

The next step was HAT to the carboxyl radical fragment to form a carboxylic acid (in the case of H_2_O_2_, the hydrogen atom is transferred to a hydroxyl radical fragment to form water). In all cases, the barriers for these reactions are very low (Table S5), suggesting that this step is not rate-determining. In some cases, it was not always clear if these reactions should be assigned as genuine synchronous HAT or if this was something more akin to sequential PT/ET or ET/PT, as discussed in the SI (Text S3), but there is no reason to expect that this would alter the conclusion that these steps are not rate-determining.

It is also important to note that the calculations revealed a great deal of complexity regarding the electronic structure of the resulting ferryl species (**5** and **8**). Briefly, the current computational methodology best describes these ferryl species as high-spin Fe(III) antiferromagnetically coupled to an oxyl radical [this electronic structure is indicated as **5′** and **8′** (Figure 4b)]. This involves promotion of an electron into the d_z2_ orbital on Fe from an Fe–O bonding molecular orbital and is in contrast to the expected electronic structure that lacks this antiferromagnetic coupling and possesses a shorter Fe–O bond (Figure 4a). These electronic structure issues are discussed in extensive detail in the SI (Text S4).

**Figure 5:**
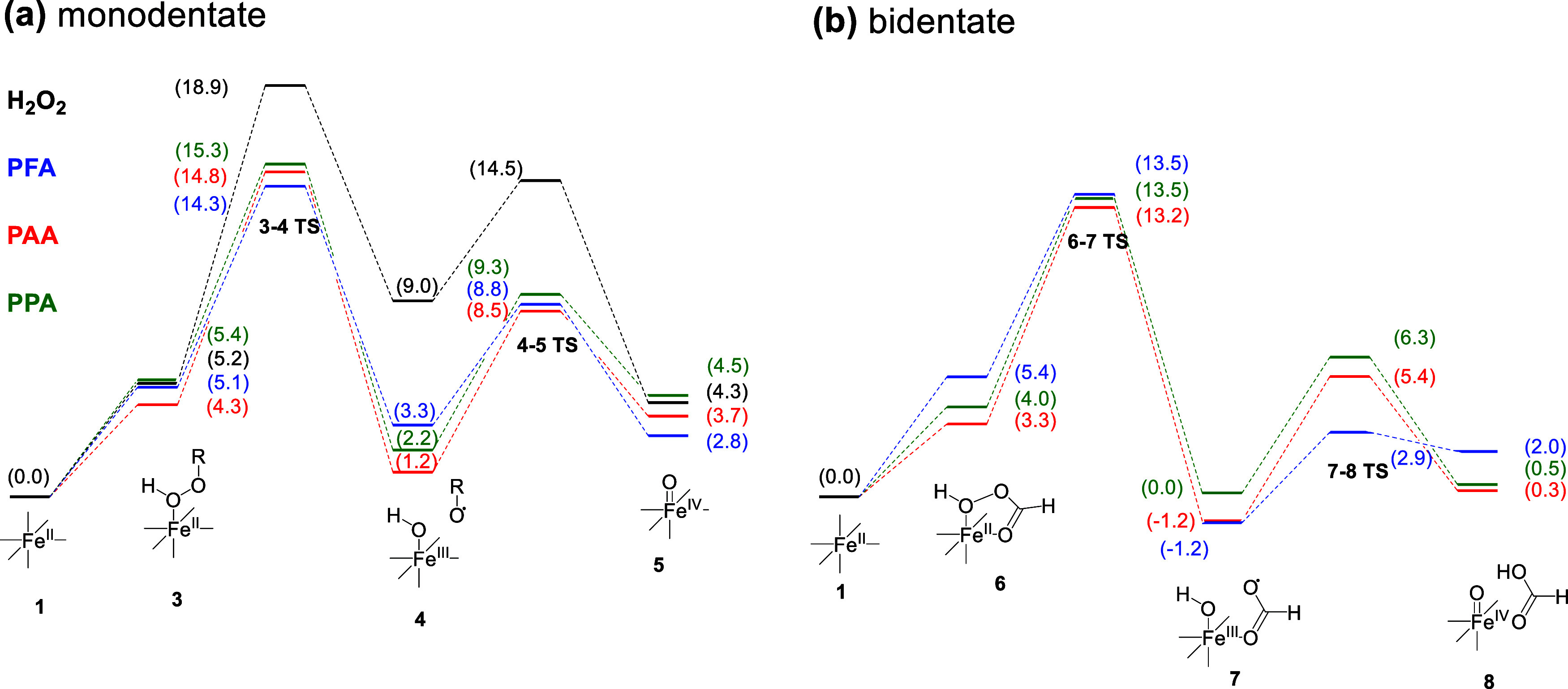
Calculated reaction coordinate diagrams for monodentate (a) and bidentate (b) pathways (energies in kcal·mol^–1^).

Regardless, a clear picture of these reactions emerges in the calculated reaction coordinate diagrams (Figure 5). The calculated mechanisms all provide low energy pathways, suggesting that they are plausible. The rate-determining step is the cleavage of the O–O bond for H_2_O_2_ and each POA. No matter the binding mode, protonation state, or nature of their peroxide activator, subsequent barriers are always lower in energy. Hence, the stronger O–O bond strength of H_2_O_2_ contributes to its experimentally observed slower reactivity when activated by Fe(II) (compared with that of POAs). While there are some differences in the barrier heights for individual steps among the POAs, the actual barrier height needs to include the energy input from binding POAs to Fe(II), which when included renders the overall activation energy comparable among all of them. In addition, the calculations demonstrate the significance of the unique bidentate mode in Fe(II)-POA reactions in aqueous solution. This chelated binding mode is slightly energetically favored and probably co-occurs with the monodentate pathway.

### Reactivity of POAs with Other Metals

To further understand POA chemistry, the decay of POAs in the presence of Co(II), Cu(II), Mn(II), and Cr(III) was investigated in batch reactors (Figure S10). The second-order rate constants were not calculated due to the complexity of these systems. For example, Co(II) and Co(III) both could contribute to POA decay, and the reaction pathways have remained controversial. In the Cu(II) systems, Cu(II) can be reduced to Cu(I) by H_2_O_2_, and hence, it is difficult to differentiate the contributions from Cu(I) and Cu(II) on POA decay.^62^ However, PFA degradation was obviously faster than that of PAA and PPA, even when its self-decay was taken into account, indicating a higher reactivity of PFA with the four tested metal ions. Despite the high reactivity of PFA, the micropollutant removal in metal-PFA systems may be limited by the fast radical decay and the strong scavenging effect of FA, like those observed in the Fe(II)-PFA process. In addition, the reactive species in other metal-POA systems are diverse and unclear. For example, Co(II)-PAA can generate Co(III), organic radicals, and possibly Co(IV).^31,63^ High-valent Mn and Cr could be produced in their corresponding AOP systems.^36,64^ Therefore, the performance of other metal-POA AOPs on the abatement of micropollutants, as well as the reactive species in other metal-PFA and metal-PPA systems, warrants further investigation.

### Environmental Significance and Implications

This study, for the first time, introduces the two simplest analogues of PAA (i.e., PFA and PPA) to metal-induced AOPs, and it systematically revealed their reaction mechanisms and decontamination efficiency in Fenton-like reactions. Herein, we highlight the high reactivity and Fe(IV) selectivity of the POAs. Relative to conventional oxidants like H_2_O_2_ and S_2_O_8_^2–^, as the POAs demonstrated about 4 orders of magnitude higher reactivity and high Fe(IV) selectivity. Compared with radicals, high-valent iron species usually selectively react with electron-rich moieties on contaminants,^59,60,65,66^ while exhibiting low reactivity toward merely aliphatic or aromatic compounds. Therefore, due to efficient Fe(IV) production, Fe(II)-POA processes could effectively target phenolic and N- and S-containing contaminants and resist the inhibition from bulk organic matter. In addition, Fe(IV) exhibits relatively low reactivity to halides, making it less likely to have its reactivity suppressed by the background matrix and lead to the formation of toxic halogenated byproducts.^21,66,67^ Hence, Fe(II)-POA processes could be more promising than other Fenton-like AOPs for decontamination in complicated water matrices through efficient Fe(IV) generation.

Overall, PFA exhibited the highest reactivity toward reductive metals; however, the removal of micropollutants could be suppressed by the inevitably coexistent FA in PFA solution. Further, the efficacy of the three POAs in the AOP may vary with the target contaminants, activation methods, and water matrices. Thus, the activation of PFA and PPA with other transition metal ions and external energy (e.g., UV light) is worthy of further investigation.

Moreover, this study also includes a step-by-step DFT evaluation of a standard mechanism for Fenton-like AOPs, providing a molecular and energetic perspective of the experimentally studied reactions. The O–O bond cleavage step is identified as the rate-limiting step for the studied systems, where Fe(II)-POA exhibited a much lower energy barrier than did Fe(II)-H_2_O_2_, consistent with experimental results. This study also highlighted the relevance of the bidentate binding pathway of POAs in the aqueous solution, a binding mode that H_2_O_2_ cannot access, and is also consistent with intermediates experimentally and computationally observed for Fe complexes with more complex ligand environments.^10,46,61^ Overall, the combined data presented in this study provide further insights into the complex mechanistic landscape of aqueous POA activation by Fe(II).

## Supporting Information Available

Methods; POA decay in the absence or presence of Fe(II) and PMSO, effect of carboxylic acids on BPA removal in Fe(II)-POA processes; further details on the DFT methodology and calculated results; validation of the KinTek Quenched Flow System using HOI-BPA reaction; degradation of BPA by Fe(II)-H2O2 with different carboxylic acids; self-decay of PFA, PAA, and PPA; degradation of PFA, PAA, and PPA in Fe(II)-POA processes; degradation of PFA, PAA, and PPA in Fe(II)-POA processes in the presence of PMSO; consumption of Fe(II) and PAA in Fe(II)-PAA processes at pH 3.0, 5.0, and 7.0; effects of FA, AA, and PA on BPA removal by Fe(II)-PAA; optimized structures of 3 (PFA) and 6 (PFA); linear relationship between experimental pKas; POA degradation in the presence of metal ions; analytical methods for HPLC-DAD; final pH values after 10 sec in the Fe(II)-POA experiments; removal of PMSO by Fe(II)-POA processes; removal of BPA by Fe(II)-POA processes (in the presence and absence of TBA); calculated reaction energetics (in kcal/mol); calculated pKas for free peroxides and metal-bound peroxides in both monodentate (3) and bidentate (6) binding modes; Mulliken spin density changes for bidentate activation of PFA; calculated energies (kcal/mol, 298.15 K); benchmarking calculations for the gas phase electronic energy reported relative to 3(H2O2) (Erel); and test set of complexes used to gauge the accuracy of pKa calculations ( PDF)
Cartesian coordinates of all the DFT-optimized structures from this study ( XYZ)

## Supplementary Material

es4c05234_si_001.pdf

es4c05234_si_002.xyz
