# Supplementary material for: Rapid and Highly Selective Fe(IV) Generation by Fe(II)-Peroxyacid Advanced Oxidation Processes: Mechanistic Investigation via Kinetics and Density Functional Theory
Source: Environ Sci Technol. 2024 Sep 14;58(38):17157–67. doi: 10.1021/acs.est.4c05234 (PMC11428173; doi:10.1021/acs.est.4c05234)
Supplement: Supplementary file 1 — es4c05234_si_001.pdf [file es4c05234_si_001.pdf]

Supporting Information

Rapid and Highly Selective Fe(IV) Generation by Fe(II)-Peroxyacid Advanced Oxidation Processes: Mechanistic Investigation via Kinetics and Density Functional Theory

Junyue Wang,[†] Juhee Kim,[†] Jiaqi Li,[†] Caroline Krall,[†] Virender K. Sharma,[*,‡] Daniel C. Ashley,[*,§] Ching-Hua Huang[*,†]

[†]School of Civil and Environmental Engineering, Georgia Institute of Technology, Atlanta, Georgia 30332, United States

[‡] School of Public Health, Texas A&M University, College Station, Texas 77843, United States

[§]Department of Chemistry and Biochemistry, Spelman College, Atlanta, Georgia 30314, United States

*Corresponding Authors.

E-mails: ching-hua.huang@ce.gatech.edu (Ching-Hua Huang); danielashley@spelman.edu (Daniel C. Ashley); vsharma@tamu.edu (Virender K. Sharma)

Numbers of Pages: 35

Numbers of Texts: 4

Numbers of Tables: 10

Numbers of Figures: 10

Numbers of References: 47

Text S1. Chemicals and Reagents

PAA solution (32% PAA and 6% H_2O_2 w/w in acetic acid and water solution) and hydrogen peroxide solution (30% H_2O_2 w/w in water) were purchased from Sigma-Aldrich (St. Louis, MO). The oxidant concentrations were determined by titration methods as described by Kim et al.[1] Additional H_2O_2 (20% of PAA, M/M) was dosed into PAA working solutions prior to all the PAA-experiments to keep the coexistent H_2O_2 concentration the same as that in PFA and PPA (57-63% of the POAs, M/M).

PFA was synthesized by mixing formic acid (2.5 mL), H_2O_2 (1.5 mL), and sulfuric acid (0.2 mL) in a 40 °C water bath for 8.0 min. PPA was synthesized by mixing propionic acid (1.0 mL) and H_2O_2 (2.0 mL), and sulfuric acid (0.2 mL) in a 40 °C water bath for 15.0 min, as described in our previous study.[2] PFA was stored at -4 °C and used within 2 h and PPA was stored at -4 °C for up to one week, where their concentrations did not change during the time periods. The POA concentrations were checked again before all the experiments. All the POA and Fe(II) working solutions were freshly prepared.

Bisphenol A (BPA), benzoic acid (BA), methyl phenyl sulfoxide (PMSO), methyl phenyl sulfone ($PMSO_2$), *tert*-butyl alcohol (TBA), sodium hydroxide (NaOH), sulfuric acid (H_2SO_4), sodium thiosulfate ($Na_2S_2O_3$), formic acid (HC(O)OH), acetic acid ($CH_3C(O)OH$), propionic acid ($CH_3CH_2C(O)OH$), potassium iodide (KI), *N,N*-diethyl-*p*-phenylenediamine (DPD) were purchased from Sigma-Aldrich or Fisher Scientific (Fair Lawn, NJ). Deionized water (DI water) (> 18 mΩ-cm) was produced from a Milli-Q water purification system (Billerica, MA).

Text S2. Density Functional Theory Methodology

All electronic structure calculations were performed using Density Functional Theory (DFT) as implemented in the Gaussian 16 electronic structure package.[3] The geometry of each molecule was optimized using B3LYP-D3[4-8] and 6-31G**[9] on all atoms except Fe, which was modeled using the SDD basis set and associated pseudopotential.[10] Frequency calculations were performed on each optimized structure to verify the nature of the stationary point (i.e., confirming it was either a minimum or transition state) and also to determine the thermochemical corrections necessary to calculate the Gibbs free energy of each molecule. The electronic energy was then determined at these optimized geometries with a single point energy calculation using PBE0-D3[11-14] and aug-cc-pVTZ[15-17] on all atoms. This energy is referred to as E_{sol}. The SMD implicit solvation model was included in all calculations (optimizations, single points, and frequencies) to account for the effect of water.[18] Because of this, the electronic energy is not a "pure" gas phase electronic energy, but a "solvated" electronic energy. All of these single point energy calculations were followed by stability calculations to ensure that the wavefunction was stable.

The thermochemical corrections for enthalpy (H_{corr}, which includes thermal effects and ZPE) and free energy (G_{corr}, which includes entropic corrections as well as H_{corr}) were used to determine the H_{sol}, TS_{sol}, and G_{sol} (Eqs S1-S3). The thermochemical corrections were calculated assuming each molecule was an ideal gas at 1 atm of pressure and 298.15 K. This did not correspond to the standard state concentration of 1 M, and so each G_{sol} required a small correction to yield G^{o}_{sol} (Eq S4).[19] The only compound that this correction was not applied to was water, which was instead corrected to be at a concentration of 55.5 M (Eq S5), which although not technically the standard state concentration is still referred to as G^{o}_{sol}. G^{o}_{sol} is used to calculate ΔG^{o} and $\Delta G^{\ddagger}$ in all cases. All calculated energies are given in Table S8. Table S4 lists the calculated ΔG^{o} and $\Delta G^{\ddagger}$ for the reactions shown in Figure 3a.

$$(\boldsymbol{S1}) \qquad E_{sol} + H_{corr} = H_{sol}$$

$$(\boldsymbol{S2}) \qquad E_{sol} + G_{corr} = G_{sol}$$

$$(\boldsymbol{S3}) \qquad G_{sol} - H_{sol} = -TS_{sol}$$

$$(\boldsymbol{S4}) \qquad G_{sol} - RT\ln(24.5) = G^{\circ}_{sol}$$

$$(\boldsymbol{S5}) \qquad G_{sol} - RT\ln(24.5 \cdot 55.5) = G^{\circ}_{sol}$$

This specific computational methodology used was chosen based on previous work by Lu et al.[18] and a small amount of additional benchmarking by us. Lu and coworkers had benchmarked their methodology by calculating the electronic energy of **4(H_2O_2)** and **5(H_2O_2)** relative to **3(H_2O_2)**, referred to presently with the numbering from the current manuscript, using a variety of density functionals and basis sets. These were then benchmarked against BD(T) energies. They ultimately chose to use PBE0-D3 for all calculations and the 6-31G* basis set on all atoms based on these results, deeming PBE0-D3 to be the most accurate functional and not seeing significant improvement on moving to the larger basis sets.

We performed a small amount of additional benchmarking as well and ultimately settled on a similar computational methodology (the most relevant of these results are given in Table S4). The SDD basis set and pseudopotential were used for geometry optimizations to keep the calculations reasonably affordable in terms of computational speed. B3LYP-D3 was used as the functional for geometry optimization simply because it did not appear to ultimately produce less accurate results and is the functional used more frequently by our lab. Electronic energies were obtained from single point energy calculations using PBE0-D3, but out of a preponderance of caution we chose to use the larger aug-cc-pVTZ basis set. As shown in Table S9, these changes still allowed for good agreement with the previously published BD(T) results. It should be noted that in Lu and coworkers' study COSMO was used to model the solvent, while we chose SMD due to its excellent ability to reproduce free energies of solvation. All benchmarking was performed in the gas phase, however.

It was critical for our discussions of speciation throughout this mechanism to have reasonable estimates of the pK_as of the potential reaction intermediates. The pK_a of a generic acid HA can be calculated if the solvated free energy of HA, A^-, and H^+ are all known. The solvated free energy of H^+, $G^o_{sol}(H^+)$, was calculated based on the estimate of its free energy of solvation (ΔG^o_{solv} = –265.9 kcal/mol).[20] The individual solvated free energies of HA and A^- can be calculated as described earlier, and the difference between the two can be referred to as ΔG^o_{deprot}. Addition of $G^o(H^+)$ to this provides ΔG^o for the deprotonation which when divided by 1.36 leads to the pK_a (this assumes a temperature of 298.15 K). This is sometimes known as the "direct" method of calculating pK_a and is shown in Eqs S6-S8.[21] Another older approach is to use a thermochemical cycle, which can yield similar results, but in cases where the

geometry is very different in solution the direct method may be advantageous.[21] Unfortunately, computational estimation of pK_as using implicit solvation models like this can often contain significant errors, and care always has to be taken when interpreting these calculated values.

$$(\boldsymbol{S6}) \qquad G^{\circ}_{sol}(A^{-}) - G^{\circ}_{sol}(HA) = \Delta G^{\circ}_{deprot}$$

$$(\boldsymbol{S7}) \qquad \Delta G^{\circ}_{deprot} + G^{\circ}_{sol}(H^{+}) = \Delta G^{\circ}_{pKa}$$

$$(\boldsymbol{S8}) \qquad \Delta G^{\circ}_{pKa} = pK_a \cdot 1.36$$

Lu and coworkers were concerned that calculating their pK_as like this would be potentially error-prone and used a different approach, which we also applied. By recognizing that ΔG^{o}_{deprot} will be proportional to ΔG^{o}_{pKa}, a calibration curve can be prepared using complexes with known pK_as. In Lu's work they were able to use this to estimate pK_as with high accuracy when compared to experiment, often within one pK_a unit.[18] We applied this technique as well, and this data is described below. The experimental pK_as of several metal aqua complexes were plotted vs the calculated ΔG^{o}_{deprot} to generate Figure S6. The linear fit from this plot could then be used to estimate the pK_a, referred to as pK_a(fit), for any complex where we had determined ΔG^{o}_{deprot}. We then used several representative test compounds (the same one used by Lu et al.) to verify the method (Table S9). We were able to estimate pK_as with relatively low errors, and so pK_a(fit) was used throughout this study instead of directly calculating pK_a. Table S5 lists the calculated pK_as relevant for the current study.

When needed, reduction potentials were calculated using Eq S9, where n is the number of electrons transferred and F is Faraday's constant.

$$(\boldsymbol{S9}) \qquad E^{\circ}(eV) = -\frac{\Delta G^{\circ}_{sol}}{nF} - 4.28$$

These potentials were reported relative to the Normal Hydrogen Electrode (NHE) by subtracting 4.28 V, the reported absolute reduction potential of NHE.[19]

Text S3 Discussion of deprotonated pathway and HAT steps

As shown in Figure 3a and discussed in the main text, an alternate reaction pathway was also considered for the deprotonated **6(PFA)**, referred to as **6a(PFA)**. The O–O bond formation for **6a(PFA)** was associated with a ΔG° and ΔG‡ of –2.4 kcal/mol and 8.4 kcal/mol respectively. To complete the mechanism HAT must occur from a bound water ligand to the metal-bound PFA fragment in **7a(PFA)**. When the PFA fragment was moved towards this water ligand during the course of a relaxed potential energy scan, it immediately triggered an electron transfer to the PFA fragment, suggesting that electron-transfer (ET) may occur in a barrierless fashion before subsequent proton-transfer (PT). The structure immediately resulting from the ET is referred to as **7b(PFA)** and corresponds to a formally Fe(IV)=O bound to performate (the ΔG° for this step was –3.4 kcal/mol). The subsequent proton transfer step for **7b(PFA)** was associated with a ΔG° and ΔG‡ of 3.4 kcal/mol and 2.8 kcal/mol respectively. Although this specific case was relatively easy to interpret in general, the HAT transition states may not have been fully synchronous in all cases or could have possibly been connected to a PT or ET step. Attempts to clarify this with Intrinsic Reaction Coordinate (IRC) calculations were, unfortunately, generally unsuccessful.

Text S4 Discussion of the electronic structure of the ferryl complexes.

The electronic structure of the ferryl species (**5** and **8**) was actually quite complex and is difficult to completely disentangle from potential methodological artifacts. The originally calculated electronic structures of **5** and **8**, the resulting products of the HAT, had α spin density on both the oxygen and Fe, but in the transition states that formed them there is β spin density on the oxygen, more so than in **4** and **7**, suggesting an increase of AF-coupled diradical character that is absent in the end product (Table S6**)**. While this isn't unheard of during a transition state, it still warranted further investigation as this could be an indicator that the transition states found are connected to a product in an alternative diradical electronic state with higher spin density on Fe.

These suspicions were confirmed explicitly for **8(PFA)**, where an IRC calculation clearly led to a resulting product structure with a large β spin density on the oxo/oxyl. This alternative electronic state (hereafter referred to with a **′**. Ex: **8′(PFA)**) could be optimized, and it was found to actually be lower in energy than the originally calculated oxo. This state was then subsequently found for the other POAs as well. Simplified electronic structures of **8** and **8′** are compared in Figure 3b. The origin of the diradical character in **8′** is promotion of an electron from a Fe–O bonding MO into the dz^2 orbital, which remains vacant for **8**. This has a significant effect on the Fe–O bond length, with it increasing by ~ 0.2 Å in **8′**. This was also seen in the heptet state of **8**, $\mathbf{8}^7$, which is essentially the ferromagnetically coupled analogue of **8′**. Both quintet structures still have radical character on the oxyl oxygen, but it arises for different reasons and has the same spin as the Fe center for **8** but opposite spin for **8′**. Note that similar results/conclusions were found for **5** as well.

While this electronic structure is not entirely implausible, it may be arising as an artifact of hybrid DFT. Hybrid DFT functionals (such as the ones used in this study) are known for over stabilizing higher spin states, which can also lead to an increase in diradical character in broken-symmetry wavefunctions. Previous work by one of the authors on a Mn(V)=O system explored this and found that while hybrid DFT was exaggerating the diradical character in the Mn–O bond (based on comparison to CASSCF calculations) it wasn't completely artifactual either.[22] It is possible then that a similar result may be true here as well, with the more realistic Fe=O bonding picture being intermediate of **8** and **8′**. In the absence of higher-

level calculations however, it is difficult to say, and such calculations are well beyond the scope of the current study.

While this diradical character was certainly more apparent for the POAs, it is actually evident for the H_2O_2 case as well. An IRC calculation was also performed on the H_2O_2 HAT transition state, and this verified that it was directly forming the same type of diradical electronic state. This suggests that the same issues were at play in Lu et al.'s published mechanism as well. Ultimately, regardless of the true nature of the electronic state of the resulting Fe(IV)=O, the diradical state (either the quintet or heptet, whichever was lowest in energy) was used for all reported values (unless noted otherwise) for the sake of consistency, given that it is the lowest energy state calculated with our current methodology.

Another consequence of the overstabilization of this diradical state may be that the preceding HAT transition states are over stabilized as well, and their actual barriers may be larger. The best way to resolve this would be to use non-hybrid functionals to characterize these steps, however Lu and coworkers have shown that non-hybrid functionals result in grievous errors when it comes to matching high-level wavefunction method energetics (BD(T) in this case). For the purposes of comparing barrier heights throughout the mechanism however, it is worth noting that similar overstabilization issues may occur in the broken-symmetry wavefunctions of the O–O bond cleavage transition states as well, so some fortuitous error cancellation may occur.

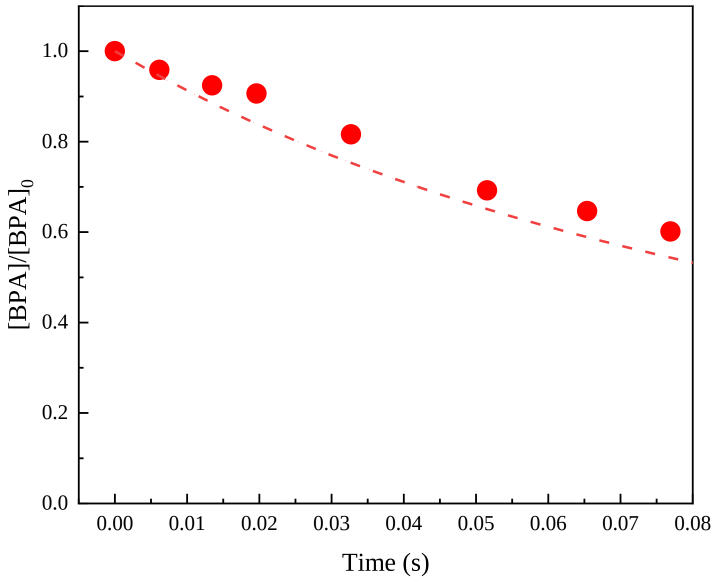

Figure S1. Validation of the KinTek Quenched Flow System using HOI-BPA reaction. Experimental conditions: $[BPA]_0$ = 50 µM, $[HOCl]_0$ = $[iodide]_0$ = 80 µM, pH = 7.0, [phosphate buffer] = 2 mM, temperature = 23 ± 2 °C. Dash line represents kinetic simulation (HOI + BPA = products, $k = 1.19\times10^5$ $M^{-1}s^{-1}$; HOCl + I^- = HOI + Cl^-, $k = 4.3\times 10^8$ $M^{-1}s^{-1}$).[23,24]

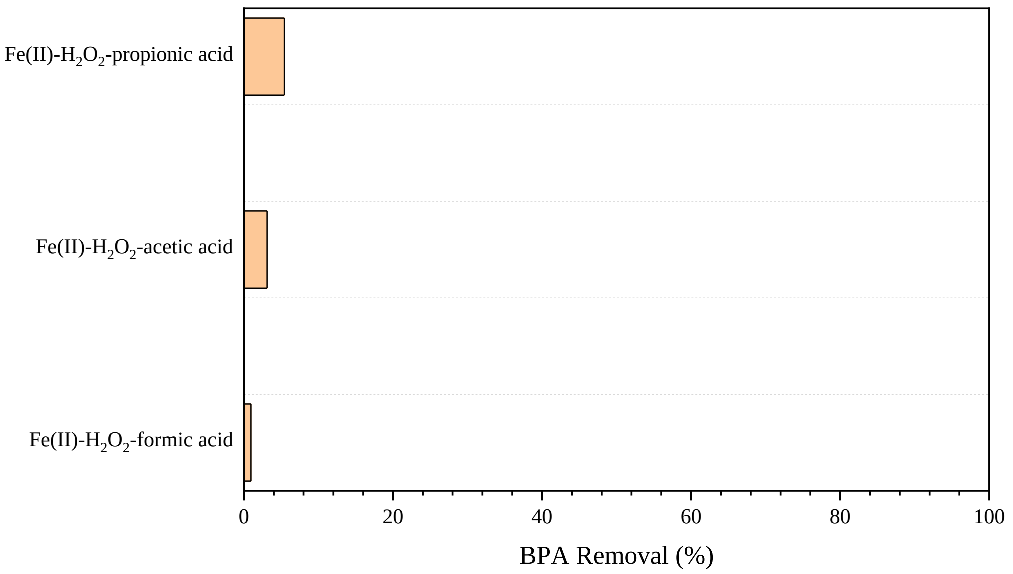

Figure S2. Degradation of BPA by Fe(II)-H_2O_2 with different carboxylic acids. Experimental conditions: $[BPA]_0$ = 10 μM, $[Fe(II)]_0$ = $[H_2O_2]_0$ = 100 μM, pH = 3.0, [carboxylic acids] = 300 μM, reaction time = 30 s.

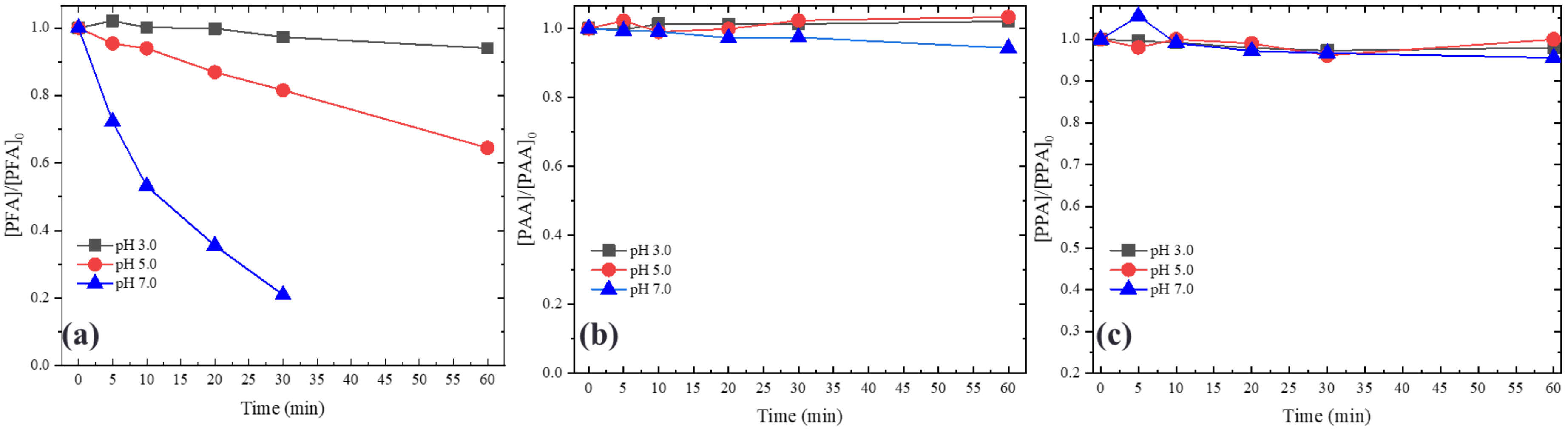

Figure S3. Self-decay of PFA (a), PAA (b), and PPA (c). $[POAs]_0$ = 100 μM, pH was not buffered but maintained at desired values by NaOH and H_2SO_4, temperature = 23 ± 2 °C.

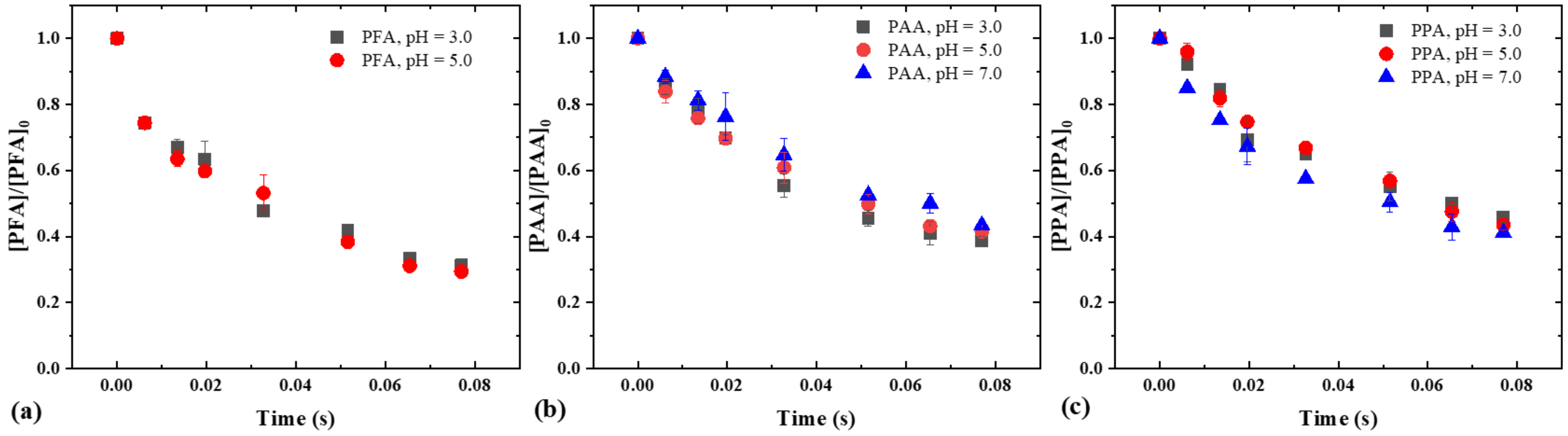

Figure S4. Degradation of PFA (a), PAA (b), and PPA (c) in Fe(II)-POA processes. Experimental conditions: $[POA]_0 = [Fe(II)]_0 = 100$ μM, [coexistent $H_2O_2]_0$ = 57-63 μM for all POAs, no buffer, temperature = 23 ± 2 °C.

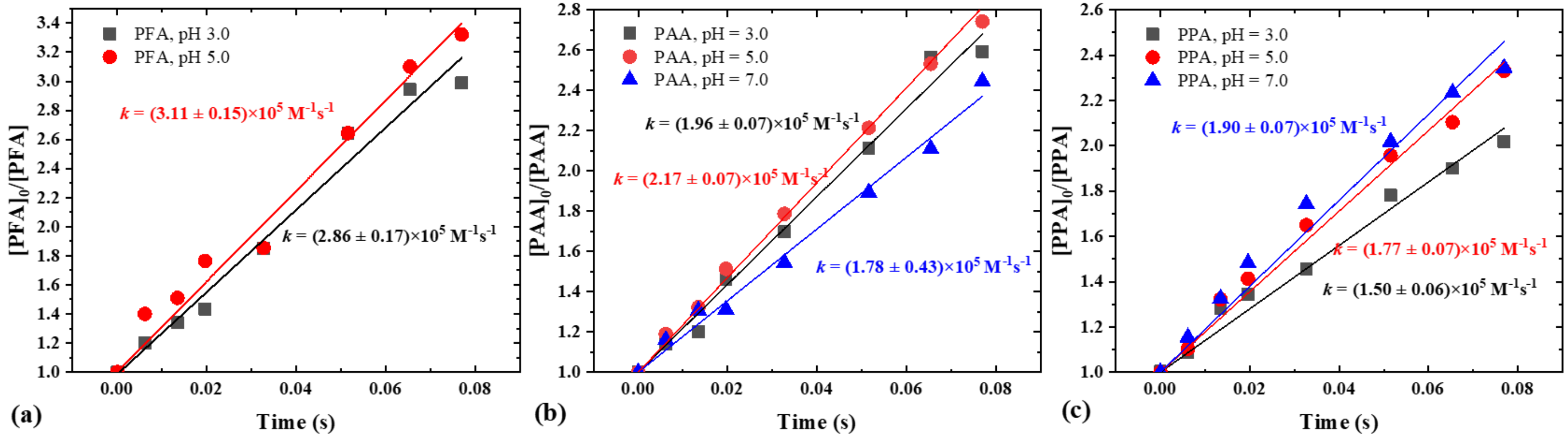

Figure S5. Degradation of PFA (a), PAA (b), and PPA (c) in Fe(II)-POA processes in the presence of PMSO. Experimental conditions: $[POA]_0 = [Fe(II)]_0 = 100$ µM, [coexistent $H_2O_2]_0 = 57$-63 µM for all POAs, no buffer, $[PMSO]_0 = 500$ µM, temperature = 23 ± 2 °C. Error bars represent standard deviations between parallel experiments.

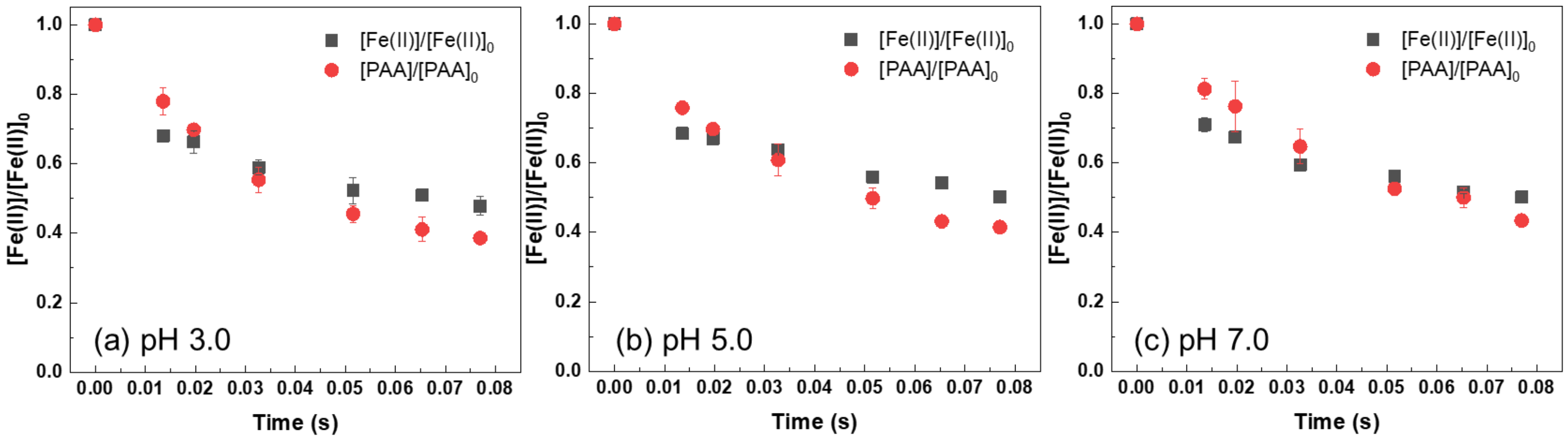

Figure S6. Consumption of Fe(II) and PAA in Fe(II)-PAA processes at pH 3.0 (a), 5.0 (b), and 7.0 (c). Experimental conditions: $[PAA]_0$ = $[Fe(II)]_0$ = 100 μM, [coexistent $H_2O_2]_0$ = 60 μM, no buffer, temperature = 23 ± 2 °C. Error bars represent standard deviations between parallel experiments.

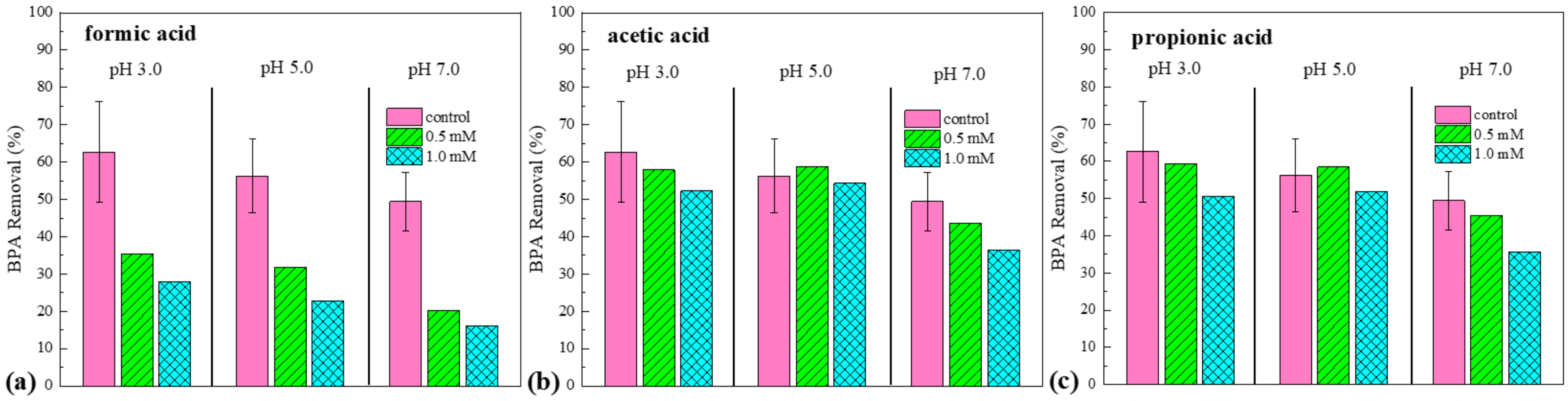

Figure S7. Effects of FA (a), AA (b), PA (c) on BPA removal by Fe(II)-PAA. Experimental conditions: $[PAA]_0 = [Fe(II)]_0 = 100$ μM, [coexistent $H_2O_2]_0 = 60$ μM, $[BPA]_0 = 10$ μM, no buffer, reaction time = 10 s, temperature = 23 ± 2 °C, pHs represent the initial values. All the solutions contained 236 μM acetic acid that coexistent with PAA, the acetic acid concentration in b represent additionally added acetic acid.

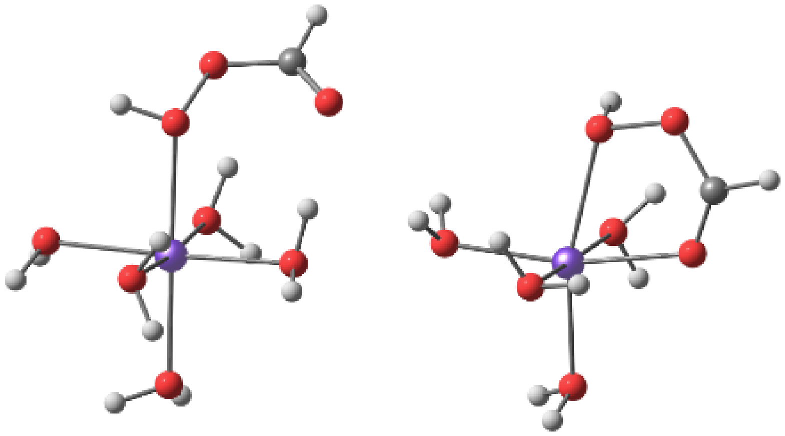

Figure S8. Optimized structures of **3 (PFA)** and **6 (PFA)** on the left and right respectively.

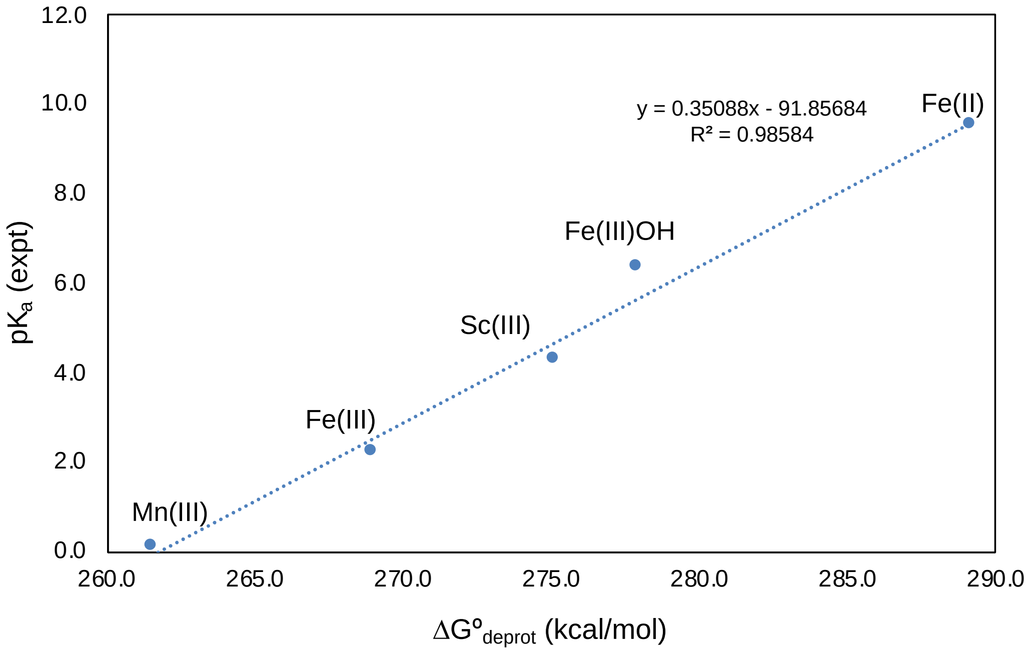

Figure S9. Linear relationship between experimental pK_as[25-27] and calculated ΔG^o_{deprot} for metal hexaaqua complexes. The deprotonation of Fe(III)OH corresponds to the 2nd pK_a of Fe(III).

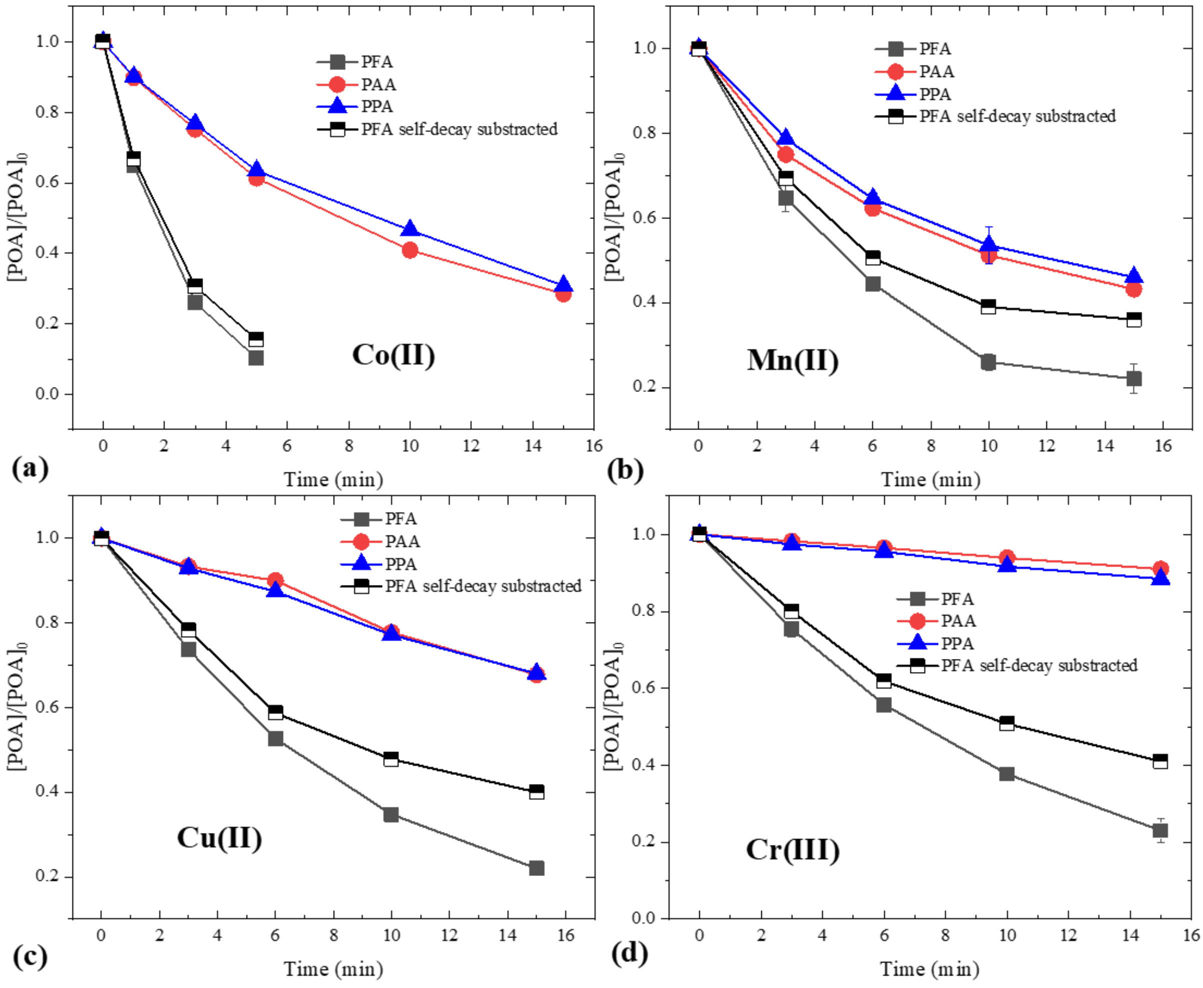

Figure S10. POA degradation in the presence of metal ions. Experimental conditions: $[POAs]_0$ = 100 μM, [coexistent $H_2O_2]_0$ = 57-63 μM for all POAs, $[Co(II)]_0$ = 10 μM, $[Mn(II)]_0$ = $[Cu(II)]_0$ = $[Cr(III)]_0$ = 2 mM, pH = 5.0 ± 0.1, pH was not buffered but monitored throughout the experiments and adjusted if necessary, temperature = 23 ± 2 °C. Error bars represent the standard deviation between parallel experiments.

Table S1. Analytical methods for HPLC-DAD

compounds	flow rate (mL/min)	solvent (H_2O:acetonitrile, v/v)	injection volume (μL)	wavelength (nm)
BPA	0.25	60/40	100	210
PMSO	0.3	80/20	60	230
$PMSO_2$	0.3	80/20	60	268
BA	0.4	80/20	100	257

Table S2. Final pHs after 10 sec in the Fe(II)-POA experiments.

POAs	$pH_0 = 3.0 \pm 0.1$	$pH_0 = 5.0 \pm 0.1$	$pH_0 = 7.0 \pm 0.1$
PFA	3.0 ± 0.1	4.3 ± 0.1	not tested
PAA	3.0 ± 0.1	3.9 ± 0.1	4.3 ± 0.1
PPA	3.0 ± 0.1	4.3 ± 0.1	4.9 ± 0.1

Note: Experimental conditions: $[POA]_0 = [Fe(II)]_0 = 100$ μM, [coexistent $H_2O_2]_0 = 57$-63 μM for all POAs, no buffer, temperature = 23 ± 2 °C

Table S3. Removal of PMSO by Fe(II)-POA processes.

POAs	pH_0	PMSO loss (μM)	$PMSO_2$ production (μM)	PMSO removal (%)	Fe(IV) contributed removal (%)	radical contributed removal (%)
PFA	3.0 ± 0.1	4.37	4.01	8.74	8.03	0.71
PFA	5.0 ± 0.1	2.01	1.30	4.03	2.60	1.42
PAA	3.0 ± 0.1	27.65	27.80	55.29	54.94	0.35
PAA	5.0 ± 0.1	12.24	8.06	24.47	16.12	8.35
PAA	7.0 ± 0.1	4.74	3.50	9.48	6.99	2.48
PPA	3.0 ± 0.1	24.77	25.55	49.53	49.53	0
PPA	5.0 ± 0.1	10.44	9.78	20.87	19.56	1.31
PPA	7.0 ± 0.1	3.01	2.04	6.02	4.08	1.94

Note: Experimental conditions: $[POA]_0 = [Fe(II)]_0 = 100$ μM, [coexistent $H_2O_2]_0 = 57$-63 μM for all POAs, $[PMSO]_0 = 50$ μM (in d), no buffer, temperature = 23 ± 2 °C, average values of duplicated experiments were shown.

Table S4. Removal of BPA by Fe(II)-POA processes (in the presence and absence of TBA).

POAs	pH_0	removal w/ TBA (50 mM) (%)	removal w/o TBA (%)	Fe(IV) contributed removal (%)	radical contributed removal (%)
PFA	3.0 ± 0.1	17.50	20.86	17.50	3.36
PFA	5.0 ± 0.1	21.92	26.75	21.92	4.83
PAA	3.0 ± 0.1	66.67	67.70	66.67	1.04
PAA	5.0 ± 0.1	49.51	56.38	49.51	6.86
PAA	7.0 ± 0.1	44.34	49.45	44.34	5.11
PPA	3.0 ± 0.1	63.24	64.32	63.24	1.08
PPA	5.0 ± 0.1	52.81	58.47	52.81	5.65
PPA	7.0 ± 0.1	49.05	52.55	49.05	3.50

Note: Experimental conditions: $[POA]_0 = [Fe(II)]_0 = 100$ μM, [coexistent $H_2O_2]_0 = 57$-63 μM for all POAs, no buffer, temperature = 23 ± 2 °C, average values of duplicated experiments were shown.

Table S5. Calculated reaction energetics (in kcal/mol)

Binding mode	Reaction	H_2O_2		PFA		PAA		PPA	
		ΔG°	ΔG‡	ΔG°	ΔG‡	ΔG°	ΔG‡	ΔG°	ΔG‡
Mono	ROOH binding (**1** to **3**)	5.2	--[a]	5.1	--[a]	4.3	--[a]	5.4	--[a]
	O–O cleavage (**3** to **4**)	3.8	13.6	–1.9	9.2	–3.1	10.6	–3.2	9.9
	HAT (**4** to **5**)	–4.8	5.5	–0.4	5.5	2.5	7.4	2.3	7.1
Bi	ROOH binding (**1** to **6**)	--[b]	--[b]	5.4	--[a]	3.3	--[a]	4.0	--[a]
	O–O cleavage (**6** to **7**)	--[b]	--[b]	–6.6	8.2	–4.5	9.9	–3.9	9.6
	HAT (**7** to **8**)	--[b]	--[b]	3.2	4.1	1.5	6.6	0.4	6.2

Note: [a]Transition state was not searched for. [b]H_2O_2 is not capable of binding in a bidentate fashion.

Table S6. Calculated pK_as for free peroxides and metal-bound peroxides in both monodentate (**3**) and bidentate (**6**) binding modes.

complex	pK_a [a]	pK_a(fit) [b]
H_2O_2 [c]	20.7	12.9
PFA [d]	12.8	9.1
PAA [e]	14.4	9.8
PPA	14.8	10.0
3 (H_2O_2)	8.8	7.2
3 (PFA)	3.0	4.4
3 (PAA)	3.2	4.5
3 (PPA)	3.6	4.7
6 (PFA)	0.4	3.2
6 (PAA)	2.1	4.0
6 (PPA)	2.1	4.0

Note: [a]pK_a calculated using Eqs S6-S8, the standard DFT methodology used throughout the study. [b]pK_a calculated using the linear regression from Figure S6. [c]Experimental pK_a = 11.6. [d]Experimental pK_a = 7.3.[28] [e]Experimental pK_a = 8.221.[29]

Table S7. Mulliken spin density changes for bidentate activation of PFA.

	7(PFA)	7-8 TS(PFA)	8(PFA)	8′(PFA)	8^7(PFA)
MSD[a] (Fe)	4.63	4.65	3.63	4.61	4.53
MSD[a] (Oxo oxygen)	–0.42	–0.93	0.28	-0.81	1.24
E_{rel}[b]	0.0	6.3	4.8	4.4	2.3

Note: [a]Mulliken Spin Density. [b]Electronic energy relative to **7 (PFA)**. All energies in kcal/mol.

Table S8. Calculated energies. (kcal/mol, 298.15 K)

Compound	E_{sol}	H_{sol}	$-TS_{sol}$	G_{sol}	G^{o}_{sol}
PFA	-166115.40	-166089.35	-20.03	-166109.38	-166107.49
PAA	-190773.27	-190728.72	-22.37	-190751.09	-190749.20
PPA	-215421.18	-215357.78	-24.50	-215382.28	-215380.39
PFA (no =O•••H bond)	-166114.82	-166088.51	-20.06	-166108.56	-166106.67
PAA (no =O•••H bond)	-190771.67	-190726.99	-23.19	-190750.18	-190748.29
PPA (no =O•••H bond)	-215420.03	-215356.58	-25.33	-215381.90	-215380.01
Performate	-165820.75	-165802.52	-19.21	-165821.73	-165819.84
Peracetate	-190476.04	-190439.44	-21.81	-190461.26	-190459.36
Perpropanoate	-215123.62	-215068.10	-23.83	-215091.93	-215090.04
Formic acid	-119003.82	-118980.15	-17.72	-118997.86	-118995.97
Acetic acid	-143660.47	-143618.51	-20.53	-143639.04	-143637.15
Propanoic acid	-168308.77	-168247.87	-22.38	-168270.25	-168268.36
Formate	-118716.52	-118701.17	-17.41	-118718.58	-118716.69
Acetate	-143369.46	-143335.63	-21.48	-143357.11	-143355.21
Propanoate	-168017.07	-167964.46	-22.30	-167986.77	-167984.87
Formate radical	-118570.79	-118557.77	-18.14	-118575.91	-118574.02
Acetate radical	-143231.03	-143197.75	-20.96	-143218.70	-143216.81
Propanoate radical	-167879.04	-167826.87	-22.92	-167849.79	-167847.89
H_2O	-47936.22	-47920.56	-13.87	-47934.43	-47930.15
•OH	-47498.41	-47491.07	-12.70	-47503.77	-47501.88
•H	-313.13	-311.65	-8.17	-319.81	-317.92
H_2O_2	-95047.28	-95028.17	-16.67	-95044.84	-95042.94
HOO^-	-94740.96	-94730.29	-16.09	-94746.38	-94744.48
HOO•	-94643.68	-94632.41	-16.30	-94648.71	-94646.81
**1**	-1080409.56	-1080304.13	-38.85	-1080342.98	-1080341.09
**2**	-1032468.56	-1032380.41	-35.05	-1032415.46	-1032413.57
**H_2O_2**					

**3(H_2O_2)**	-1127519.80	-1127410.80	-39.75	-1127450.54	-1127448.65
**3-4 TS(H_2O_2)**	-1127506.33	-1127399.26	-37.65	-1127436.91	-1127435.02
**3a(H_2O_2)**	-1127229.33	-1127128.68	-39.66	-1127168.34	-1127166.44
**4(H_2O_2)**	-1127514.13	-1127407.34	-39.39	-1127446.73	-1127444.84
**4-5 TS(H_2O_2)**	-1127509.03	-1127404.62	-36.65	-1127441.27	-1127439.38
**5(H_2O_2)**	-1127523.13	-1127414.06	-37.17	-1127451.23	-1127449.33
**5^7(H_2O_2)**	-1127521.10	-1127412.84	-37.67	-1127450.51	-1127448.62
**5′(H_2O_2)**	-1127522.61	-1127413.80	-37.68	-1127451.48	-1127449.59
**PFA**					
**3(PFA)**	-1198588.31	-1198472.63	-42.57	-1198515.19	-1198513.30
**3-4 TS(PFA)**	-1198580.48	-1198466.04	-39.99	-1198506.03	-1198504.14
**3a(PFA)**	-1198306.54	-1198199.02	-41.81	-1198240.84	-1198238.94
**4(PFA)**	-1198592.57	-1198477.42	-39.62	-1198517.05	-1198515.15
**4-5 TS(PFA)**	-1198586.38	-1198473.32	-38.24	-1198511.56	-1198509.67
**5(PFA)**	-1198593.92	-1198476.18	-38.88	-1198515.06	-1198513.17
**5^7(PFA)**	-1198594.07	-1198478.05	-39.42	-1198517.48	-1198515.58
**5′(PFA)**	-1198593.72	-1198477.45	-39.36	-1198516.81	-1198514.92
**6(PFA)**	-1150644.83	-1150546.21	-38.58	-1150584.78	-1150582.89
**6-7 TS(PFA)**	-1150636.50	-1150539.60	-37.02	-1150576.62	-1150574.72
**6a(PFA)**	-1150365.42	-1150275.39	-38.61	-1150314.00	-1150312.11
**6a-7a TS(PFA)**	-1150357.59	-1150268.27	-37.34	-1150305.61	-1150303.71
**7(PFA)**	-1150653.14	-1150555.31	-36.07	-1150591.38	-1150589.49
**7a(PFA)**	-1150369.59	-1150279.32	-37.06	-1150316.38	-1150314.48
**7b′(PFA)**[a]	-1150373.75	-1150283.44	-36.42	-1150319.86	-1150317.97
**7b(PFA)**[a]	-1150375.05	-1150284.11	-35.67	-1150319.79	-1150317.89
**7-8 TS(PFA)**	-1150646.84	-1150552.07	-35.20	-1150587.28	-1150585.38
**7b-8a TS(PFA)**[a,b]	-1150370.71	-1150282.50	-34.45	-1150316.94	-1150315.05
**8(PFA)**	-1150648.37	-1150548.44	-35.39	-1150583.83	-1150581.94
**8^7(PFA)**	-1150650.86	-1150552.33	-35.86	-1150588.19	-1150586.29

**8′(PFA)**	-1150648.77	-1150550.30	-35.94	-1150586.25	-1150584.35
**8a(PFA)**	-1150371.03	-1150280.08	-36.35	-1150316.43	-1150314.54
**8a^{7}(PFA)**	-1150367.08	-1150277.73	-36.35	-1150314.08	-1150312.18
**8a′(PFA)**	-1150368.06	-1150278.87	-36.29	-1150315.16	-1150313.26
**PAA**					
**3(PAA)**	-1223245.98	-1223111.94	-45.82	-1223157.76	-1223155.86
**3-4 TS(PAA)**	-1223237.26	-1223104.36	-42.82	-1223147.18	-1223145.29
**3a(PAA)**	-1222963.16	-1222837.12	-43.47	-1222880.59	-1222878.69
**4(PAA)**	-1223249.13	-1223115.90	-44.95	-1223160.84	-1223158.95
**4-5 TS(PAA)**	-1223242.95	-1223111.65	-41.83	-1223153.48	-1223151.58
**5(PAA)**	-1223250.75	-1223115.02	-42.01	-1223157.02	-1223155.13
**5^{7}(PAA)**	-1223251.00	-1223116.62	-41.73	-1223158.35	-1223156.45
**5′(PAA)**	-1223249.36	-1223114.24	-41.76	-1223156.00	-1223154.10
**6(PAA)**	-1175303.44	-1175186.64	-41.94	-1175228.59	-1175226.69
**6-7 TS(PAA)**	-1175294.35	-1175179.15	-39.52	-1175218.67	-1175216.78
**6a(PAA)**	-1175022.34	-1174914.06	-41.87	-1174955.94	-1174954.04
**7(PAA)**	-1175310.08	-1175193.92	-39.18	-1175233.10	-1175231.20
**7-8 TS(PAA)**	-1175303.73	-1175189.86	-36.62	-1175226.49	-1175224.59
**8(PAA)**	-1175306.93	-1175188.60	-37.66	-1175226.26	-1175224.37
**8^{7}(PAA)**	-1175308.69	-1175192.14	-39.46	-1175231.60	-1175229.71
**8′(PAA)**	-1175305.15	-1175188.77	-39.42	-1175228.19	-1175226.29
**PPA**					
**3(PPA)**	-1247894.16	-1247741.13	-46.67	-1247787.80	-1247785.90
**3-4 TS(PPA)**	-1247885.57	-1247733.73	-44.21	-1247777.94	-1247776.04
**3a(PPA)**	-1247610.97	-1247466.26	-46.44	-1247512.70	-1247510.80
**4(PPA)**	-1247897.42	-1247745.14	-45.84	-1247790.98	-1247789.09
**4-5 TS(PPA)**	-1247891.08	-1247740.74	-43.16	-1247783.90	-1247782.01
**5(PPA)**	-1247898.18	-1247743.66	-44.80	-1247788.45	-1247786.56
**5^{7}(PPA)**	-1247898.50	-1247744.91	-43.76	-1247788.67	-1247786.78

**5′(PPA)**	-1247898.19	-1247745.16	-42.01	-1247787.17	-1247785.27
**6(PPA)**	-1199951.45	-1199815.71	-43.39	-1199859.10	-1199857.20
**6-7 TS(PPA)**	-1199942.40	-1199807.87	-41.67	-1199849.54	-1199847.64
**6a(PPA)**	-1199670.32	-1199542.99	-43.03	-1199586.02	-1199584.13
**7(PPA)**	-1199958.17	-1199822.75	-40.28	-1199863.02	-1199861.13
**7-8 TS(PPA)**	-1199951.36	-1199818.43	-38.37	-1199856.79	-1199854.90
**8(PPA)**	-1199954.68	-1199817.29	-39.25	-1199856.54	-1199854.64
**8^{7}(PPA)**	-1199957.09	-1199821.71	-40.87	-1199862.58	-1199860.69
**8′(PPA)**	-1199952.25	-1199816.94	-41.06	-1199858.00	-1199856.11
**pK_a benchmarking molecules[c]**					
Sc(III)	-764759.77	-764654.74	-36.63	-764691.36	-764689.47
Sc(III)OH	-764476.81	-764379.83	-36.38	-764416.22	-764414.32
Mn(III)	-1009565.14	-1009457.13	-33.68	-1009490.81	-1009488.91
Mn(III)OH	-1009293.04	-1009194.42	-34.85	-1009229.27	-1009227.37
Fe(II)	-1080371.48	-1080264.36	-34.77	-1080299.14	-1080297.24
Fe(II)OH	-1080073.18	-1079975.45	-34.50	-1080009.94	-1080008.05
Fe(III)	-1080291.19	-1080184.17	-34.69	-1080218.86	-1080216.97
Fe(III)OH	-1080011.71	-1079913.66	-36.20	-1079949.86	-1079947.96
Fe(III)$(OH)_2$	-1079725.20	-1079635.91	-36.04	-1079671.95	-1079670.05
Ti(III)	-820406.54	-820301.40	-37.71	-820339.10	-820337.21
Ti(III)OH	-820129.50	-820031.89	-35.23	-820067.12	-820065.22
V(III)	-879703.87	-879596.89	-35.52	-879632.41	-879630.51
V(III)OH	-879426.76	-879328.54	-35.42	-879363.96	-879362.06
Cr(III)	-942742.54	-942634.83	-33.88	-942668.71	-942666.81
Cr(III)OH	-942460.96	-942361.75	-33.55	-942395.30	-942393.41
Co(II)	-1155110.51	-1155003.88	-36.29	-1155040.17	-1155038.28
Co(II)OH	-1154812.41	-1154715.38	-38.06	-1154753.44	-1154751.55
Co(III)	-1154954.44	-1154842.19	-30.19	-1154872.39	-1154870.49
Co(III)OH	-1154677.90	-1154576.69	-31.35	-1154608.03	-1154606.13

Ni(II)	-1233882.81	-1233775.66	-36.20	-1233811.87	-1233809.97
Ni(II)OH	-1233584.93	-1233486.63	-35.54	-1233522.17	-1233520.28
V(IV)O	-879041.10	-878949.16	-33.34	-878982.50	-878980.60
V(IV)(O)(OH)	-878757.55	-878674.00	-33.13	-878707.12	-878705.23

Note: [a]**7b(PFA)** refers to **7a(PFA)** where an electron has already transferred to the PFA fragment. This means that it is essentially already formally Fe(IV)=O bound to performate. [b]This transition state corresponds to transferring a proton from a bound aqua ligand to the bound performate anion. [c]These compounds are all six-coordinate, where each ligand can be assumed to be H_2O unless it is explicitly indicated otherwise. The formal oxidation state of each metal is specified.

Table S9. Benchmarking calculations for the gas phase electronic energy reported relative to **3(H_2O_2)** (E_{rel}).

Methodology (GO)	Methodology (SP)	E_{rel} **4(H_2O_2)**	E_{rel} **5(H_2O_2)**
[a]B3LYP-D3<br>6-31++G**	BD(T)<br>def2-TZVP	10.7	–1.6
[a]B3LYP-D3<br>6-31++G**	B3LYP-D3<br>def2-TZVP	10.8	–5.9
[a]B3LYP-D3<br>6-31++G**	PBE0-D3<br>def2-TZVP	12.6	0.7
B3LYP-D3<br>6-31G**	B3LYP-D3<br>def2-TZVP	10.6	–6.1
B3LYP-D3<br>6-31G**<br>[b]SDD (Fe)	B3LYP-D3<br>def2-TZVP	11.0	–5.7
PBE0-D3<br>6-31G**<br>[b]SDD (Fe)	PBE0-D3<br>aug-cc-pVTZ	12.1	0.1
B3LYP-D3<br>6-31G**<br>[b]SDD (Fe)	PBE0-D3<br>aug-cc-pVTZ	12.0	–0.1

Note: The indicated basis sets were used on all atoms unless indicated otherwise. [a]These results are taken directly from Lu and coworkers previously published results.[18] [b]The SDD basis set and associated pseudopotential was used on Fe.

Table S10. Test set of complexes used to gauge the accuracy of pK_a calculations.

Complex	pK_a (calc)[a]	pK_a (fit)[b]	pK_a (expt)
$[Ti^{III}(H_2O)_6]^{3+}$	1.3	3.6	2.15[30]
$[V^{III}(H_2O)_6]^{3+}$	-1.3	2.3	2.6[31]
$[Cr^{III}(H_2O)_6]^{3+}$	2.3	4.1	3.7[32]
$[Co^{II}(H_2O)_6]^{2+}$	12.1	8.8	9.7[32]
$[Co^{III}(H_2O)_6]^{3+}$	-4.3	0.9	0.5[32]
$[Ni^{II}(H_2O)_6]^{2+}$	14.3	9.8	9.9[33]
$[V^{IV}(O)H_2O)_5]^{2+}$	3.7	4.8	5.3-6.0[34-37]

Note: [a]pK_a calculated using Eqs S6-S8, the standard DFT methodology used throughout the study. [b]pK_a calculated using the linear regression from Figure S6.

References:

1. Kim, J.; Zhang, T.; Liu, W.; Du, P.; Dobson, J. T.; Huang, C.-H., Advanced Oxidation Process with Peracetic Acid and Fe(II) for Contaminant Degradation. *Environ Sci Technol* **2019,** *53*, (22), 13312-13322.
2. Wang, J.; Chen, W.; Wang, T.; Reid, E.; Krall, C.; Kim, J.; Zhang, T.; Xie, X.; Huang, C.-H., Bacteria and Virus Inactivation: Relative Efficacy and Mechanisms of Peroxyacids and Chlor(am)ine. *Environ Sci Technol* **2023,** *57*, (47), 18710-18721.
3. Gaussian 16, R. C.; Frisch, M. J.; Trucks, G. W.; Schlegel, H. B.; Scuseria, G. E.; Robb, M. A.; Cheeseman, J. R.; Scalmani, G.; Barone, V.; Petersson, G. A.; Nakatsuji, H.; Li, X.; Caricato, M.; Marenich, A. V.; Bloino, J.; Janesko, B. G.; Gomperts, R.; Mennucci, B.; Hratchian, H. P.; Ortiz, J. V.; Izmaylov, A. F.; Sonnenberg, J. L.; Williams-Young, D.; Ding, F.; Lipparini, F.; Egidi, F.; Goings, J.; Peng, B.; Petrone, A.; Henderson, T.; Ranasinghe, D.; Zakrzewski, V. G.; Gao, J.; Rega, N.; Zheng, G.; Liang, W.; Hada, M.; Ehara, M.; Toyota, K.; Fukuda, R.; Hasegawa, J.; Ishida, M.; Nakajima, T.; Honda, Y.; Kitao, O.; Nakai, H.; Vreven, T.; Throssell, K.; Montgomery Jr., J. A.; Peralta, J. E.; Ogliaro, F.; Bearpark, M. J.; Heyd, J. J.; Brothers, E. N.; Kudin, K. N.; Staroverov, V. N.; Keith, T. A.; Kobayashi, R.; Normand, J.; Raghavachari, K.; Rendell, A. P.; Burant, J. C.; Iyengar, S. S.; Tomasi, J.; Cossi, M.; Millam, J. M.; Klene, M.; Adamo, C.; Cammi, R.; Ochterski, J. W.; Martin, R. L.; Morokuma, K.; Farkas, O.; Foresman, J. B.; Fox, D. J., Gaussian, Inc., Wallingford CT, 2019. *Gaussian 16*.
4. Becke, A. D., Density-functional exchange-energy approximation with correct asymptotic behavior. *Physical Review A* **1988,** *38*, (6), 3098-3100.
5. Becke, A. D., Density-functional thermochemistry. III. The role of exact exchange. *The Journal of Chemical Physics* **1993,** *98*, 5648-5652.
6. Becke, A. D., A new mixing of Hartree–Fock and local density-functional theories. *Journal of Chemical Physics* **1993,** *98*, (2), 1372.
7. Grimme, S.; Antony, J.; Ehrlich, S.; Krieg, H., A consistent and accurate ab initio parametrization of density functional dispersion correction (DFT-D) for the 94 elements H-Pu. *The Journal of Chemical Physics* **2010,** *132*, (15), 154104.
8. Lee, C.; Yang, W.; Parr, R. G., Development of the Colle-Salvetti correlation-energy formula into a functional of the electron density. *Physical Review B* **1988,** *37*, (2), 785-789.
9. Krishnan, R.; Binkley, J. S.; Seeger, R.; Pople, J. A., Self□consistent molecular orbital methods. XX. A basis set for correlated wave functions. *The Journal of Chemical Physics* **1980,** *72*, (1), 650-654.
10. Dolg, M.; Wedig, U.; Stoll, H.; Preuss, H., Energy-adjusted ab initio pseudopotentials for the first row transition elements. *Journal of Chemical Physics* **1987,** *86*, (2), 866-872.
11. Adamo, C.; Barone, V., Toward reliable density functional methods without adjustable parameters: The PBE0 model. *The Journal of Chemical Physics* **1999,** *110*, (13), 6158-6170.
12. Ernzerhof, M.; Scuseria, G. E., Assessment of the Perdew–Burke–Ernzerhof exchange-correlation functional. *The Journal of Chemical Physics* **1999,** *110*, (11), 5029-5036.
13. Perdew, J. P.; Burke, K.; Ernzerhof, M., Generalized Gradient Approximation Made Simple. *Phys. Rev. Lett.* **1996,** *77*, (18), 3865–3868.
14. Perdew, J. P.; Burke, K.; Ernzerhof, M., Generalized Gradient Approximation Made Simple [Phys. Rev. Lett. 77, 3865 (1996)]. *Phys. Rev. Lett.* **1997,** *78*, (7), 1396–1396.
15. Balabanov, N. B.; Peterson, K. A., Systematically convergent basis sets for transition metals. I. All-electron correlation consistent basis sets for the 3d elements Sc–Zn. *The Journal of Chemical Physics*

2005, *123*, (6), 064107.
16. Dunning, T. H., Jr., Gaussian basis sets for use in correlated molecular calculations. I. The atoms boron through neon and hydrogen. *The Journal of Chemical Physics* **1989,** *90*, (2), 1007-1023.
17. Kendall, R. A.; Dunning, T. H., Jr.; Harrison, R. J., Electron affinities of the first□row atoms revisited. Systematic basis sets and wave functions. *The Journal of Chemical Physics* **1992,** *96*, (9), 6796-6806.
18. Lu, H.-F.; Chen, H.-F.; Kao, C.-L.; Chao, I.; Chen, H.-Y., A computational study of the Fenton reaction in different pH ranges. *Phys. Chem. Chem. Phys.* **2018,** *20*, (35), 22890-22901.
19. Cramer, C. J., *Essentials of Computational Chemistry: Theories and Models.* 2nd ed.; 2004.
20. Kelly, C. P.; Cramer, C. J.; Truhlar, D. G., Aqueous Solvation Free Energies of Ions and Ion−Water Clusters Based on an Accurate Value for the Absolute Aqueous Solvation Free Energy of the Proton. *The Journal of Physical Chemistry B* **2006,** *110*, (32), 16066-16081.
21. Lian, P.; Johnston, R. C.; Parks, J. M.; Smith, J. C., Quantum Chemical Calculation of pKas of Environmentally Relevant Functional Groups: Carboxylic Acids, Amines, and Thiols in Aqueous Solution. *The Journal of Physical Chemistry A* **2018,** *122*, (17), 4366-4374.
22. Ashley, D. C.; Baik, M.-H., The Electronic Structure of [Mn(V)═O]: What is the Connection between Oxyl Radical Character, Physical Oxidation State, and Reactivity? *ACS Catalysis* **2016,** *6*, (10), 7202-7216.
23. Wang, X. S.; Liu, Y. L.; Xu, S. Y.; Zhang, J.; Li, J.; Song, H.; Zhang, Z. X.; Wang, L.; Ma, J., Ferrate Oxidation of Phenolic Compounds in Iodine-Containing Water: Control of Iodinated Aromatic Products. *Environ Sci Technol* **2020,** *54*, (3), 1827-1836.
24. Wang, J.; Xu, J.; Kim, J.; Huang, C.-H., Mechanistic Insight for Disinfection Byproduct Formation Potential of Peracetic Acid and Performic Acid in Halide-Containing Water. *Environ Sci Technol* **2023,** *57*, (47), 18898-18908.
25. Lente, G.; Fábián, I., New Reaction Path in the Dissociation of the $Fe_2(\mu\text{-}OH)_2(H_2O)_8^{4+}$ Complex. *Inorganic Chemistry* **1999,** *38*, (3), 603-605.
26. Li, J.; Fisher, C. L.; Chen, J. L.; Bashford, D.; Noodleman, L., Calculation of Redox Potentials and pKa Values of Hydrated Transition Metal Cations by a Combined Density Functional and Continuum Dielectric Theory. *Inorganic Chemistry* **1996,** *35*, (16), 4694-4702.
27. Rustad, J. R.; Dixon, D. A.; Rosso, K. M.; Felmy, A. R., Trivalent Ion Hydrolysis Reactions: A Linear Free-Energy Relationship Based on Density Functional Electronic Structure Calculations. *Journal of the American Chemical Society* **1999,** *121*, (13), 3234-3235.
28. Santacesaria, E.; Russo, V.; Tesser, R.; Turco, R.; Di Serio, M., Kinetics of Performic Acid Synthesis and Decomposition. *Ind Eng Chem Res* **2017,** *56*, (45), 12940-12952.
29. Cheng, C.; Li, H.; Wang, J.; Wang, H.; Yang, X., A review of measurement methods for peracetic acid (PAA). *Frontiers of Environmental Science & Engineering* **2020,** *14*, (5), 87.
30. Chaudhuri, P.; Diebler, H., Kinetics and equilibria of the interaction of titanium(III) with oxalic acid. *J. Chem. Soc., Dalton Trans.* **1977**, (6), 596-601.
31. Kallies, B.; Meier, R., Electronic Structure of 3d $[M(H_2O)_6]^{3+}$ Ions from Sc^{III} to Fe^{III}: A Quantum Mechanical Study Based on DFT Computations and Natural Bond Orbital Analyses. *Inorganic Chemistry* **2001,** *40*, (13), 3101-3112.
32. Hawkes, S. J., All Positive Ions Give Acid Solutions in Water. *Journal of Chemical Education* **1996,** *73*, (6), 516.
33. Collini, D.; De Biani, F. F.; Fedi, S.; Femoni, C.; Kaswalder, F.; Iapalucci, M. C.; Longoni, G.;

Tiozzo, C.; Zacchini, S.; Zanello, P., Synthesis and Electrochemistry of New Rh-Centered and Conjuncto Rhodium Carbonyl Clusters. X-ray Structure of $[NEt_4]_3[Rh_{15}(CO)_{27}]$, $[NEt_4]_3[Rh_{15}(CO)_{25}(MeCN)_2]\cdot 2MeCN$, and $[NEt_4]_3[Rh_{17}(CO)_{37}]$. *Inorganic Chemistry* **2007,** *46*, (19), 7971-7981.
34. Wilkinson, G.; Gillard, R. D.; McCleverty, J. A., *In Comprehensive Coordination Chemistry*. 1987.
35. Francavilla, J.; Chasteen, N. D., Hydroxide effects on the electron paramagnetic resonance spectrum of aqueous vanadyl(IV) ion. *Inorganic Chemistry* **1975,** *14*, (11), 2860-2862.
36. Henry, R. P.; Mitchell, P. C. H.; Prue, J. E., Hydrolysis of the oxovanadium(IV) ion and the stability of its complexes with the 1,2-dihydroxybenzenato(2–) ion. *J. Chem. Soc., Dalton Trans.* **1973**, (11), 1156-1159.
37. Rossotti, F. J. C.; Rossotti, H. S., Studies on the Hydrolysis of Metal Ions. XII. The Hydrolysis of the Vanadium (IV) ion. *Acta Chemica Scandinavica* **1955,** *9*, 1177-1192.
